# Comparative efficacy and mechanistic insights of non-invasive neuromodulation and motor rehabilitation on functional reorganization of the supplementary motor area in subacute stroke: a narrative review

**DOI:** 10.3389/fphys.2026.1744260

**Published:** 2026-04-02

**Authors:** Yun-Shan Zhang, Ying-Dong Li, Cai-Xia Ou, Zi-Ting Bi, Jing-Hua Xiao, Jia-Mei Zhang, Jing-Xue Wei, Jian-Wen Xu, Lang Huang

**Affiliations:** 1 Department of Rehabilitation Medicine, The First Affiliated Hospital of Guangxi Medical University, Nanning, China; 2 Department of Rehabilitation Medicine, The Second Affiliated Hospital of Guangxi Medical University, Nanning, China; 3 Department of Rehabilitation Medicine, Guangxi International Zhuang Medicine Hospital, Nanning, China

**Keywords:** subacute stroke, supplementary motor area, non-invasive neuromodulation, motor rehabilitation, functional reorganization, neuroplasticity

## Abstract

Subacute stroke patients frequently experience significant motor impairment. The supplementary motor area (SMA), a critical hub for motor control and functional brain reorganization, plays a pivotal role in recovery. However, traditional interventions such as drug therapy and conventional physical therapy often lack the spatial precision and causal efficacy required for directly and accurately remodeling specific dysfunctional circuits like the SMA in subacute stroke, which results in key challenges in current rehabilitation practice for correcting specific network imbalances and efficiently inducing task-related plasticity. This narrative review elucidates how two advanced, mechanism-driven strategies address these challenges. Non-invasive neuromodulation provides a precise top-down intervention method that can directly regulate the cortical excitability of SMA and its related networks, correcting pathological network imbalances, which is unmatched by traditional methods. In contrast, motor rehabilitation provides a powerful bottom-up, experience-dependent intervention that drives Hebbian plasticity through intensive, task-oriented training, specifically enhancing SMA activation and functional connectivity. Crucially, the major innovation lies in their strategic combination. Non-invasive neuromodulation primes the brain network for learning, while motor rehabilitation consolidates the induced plasticity, thereby yielding synergistic effects that maximize functional recovery. This review synthesizes current evidence on the efficacy and mechanisms of these interventions in promoting SMA reorganization following subacute stroke, analyzing their impacts on network modulation, neuroimaging correlates, and clinical outcomes. By integrating foundational research and clinical insights, it aims to establish a theoretical framework for refining precision, network-targeted rehabilitation strategies for post-stroke motor deficits in the subacute phase.

## 1 Introduction

Stroke is a major global health challenge, attracting widespread attention due to its high incidence, high disability rate, high recurrence rate, high mortality rate, and heavy economic burden (Avan et al., 2019; Huang et al., 2025). With advances in acute stroke medical management, patients’ survival rates have significantly improved (Phipps and Cronin, 2020). However, many survivors enter the subacute phase with severe functional impairments (Meijer et al., 2003; Esht et al., 2024). These impairments mainly include motor deficits, such as hemiparesis, gait abnormalities, and impaired coordination, which substantially compromise activities of daily living and overall quality of life (Ceradini et al., 2024; Esht et al., 2024). The subacute phase of stroke, typically defined as the period from 1 to 6 months post-onset, is characterized by heightened neural plasticity and active functional reorganization, representing a critical window for effective rehabilitation and functional recovery (Larsen et al., 2018; Bruyn et al., 2023; Han et al., 2024). This reorganization process involves not only the primary motor cortex (M1) but also extends to higher-order motor regions, among which the supplementary motor area (SMA) plays a central role (Wu et al., 2025; Zheng et al., 2025). The SMA, situated in the medial frontal lobe, is primarily responsible for motor planning, sequence learning, bimanual coordination, and error monitoring, with its functional activity being closely linked to complex motor behaviors, such as gait control and fine motor execution (Tanji, 2001; Dali et al., 2022; Zheng et al., 2025). After a stroke, functional alterations in the SMA, including compensatory hyperactivation or pathological hypoactivation, reflect the brain’s endeavor to reorganize motor function and correlate with the extent of motor recovery (Pantano et al., 1996; Braaß et al., 2023). Therefore, targeted interventions for the SMA in subacute stroke have become a focus area in rehabilitation research.

While traditional rehabilitation methods such as passive or isolated physical therapy provide essential foundational support, their approaches are often too general and may not fully exploit the specific neural plasticity mechanisms critical during the subacute phase, particularly for higher-order motor areas like the SMA (Lazaridou et al., 2013; Zhao et al., 2024). These methods primarily focus on compensatory strategies and gross functional retraining, which might not sufficiently drive the precise, use-dependent cortical reorganization necessary for optimal recovery of complex motor functions (Lazaridou et al., 2013; Zhao et al., 2024). Similarly, the action of conventional drug therapies like neurotrophic agents is non-focal, limiting their ability to engage and remodel the specific neural networks that are crucially impaired (Vajda, 2002; Cook et al., 2017). Therefore, rehabilitation strategies need to evolve continuously, moving beyond traditional methods and exploring more targeted interventions (Bolognini et al., 2016; Xiong et al., 2022). Non-invasive neuromodulation techniques, such as repetitive transcranial magnetic stimulation (rTMS) and transcranial direct current stimulation (tDCS), have attracted significant attention due to their ability to directly modulate cortical excitability and promote neuroplasticity (Klomjai et al., 2015; Ding et al., 2022; Davidson et al., 2024). These techniques offer a unique advantage over traditional methods by enabling precise, targeted modulation of cortical or SMA network excitability, thereby directly influencing functional reorganization and restoring interhemispheric balance to create favorable neurophysiological conditions for recovery that are difficult to achieve with conventional therapy alone (Ding et al., 2022; Davidson et al., 2024). Concurrently, motor rehabilitation paradigms, such as robot-assisted training and task-oriented training, represent a significant evolution from traditional approaches. A defining characteristic of these paradigms is their motor training modality, which emphasizes high intensity, repetition, and a task-oriented nature, designed to leverage principles of motor learning and use-dependent plasticity (Bonanno et al., 2022; Afridi et al., 2023). This task-oriented framework appears particularly synergistic with the functions of the SMA, as it engages neural circuits involved in motor planning, sequence execution, and skill acquisition, domains in which the SMA plays a predominant role (Bonanno et al., 2022; Afridi et al., 2023). Through the repeated practice of functionally relevant tasks, these interventions may more effectively stimulate and guide the reorganization of SMA networks, thereby promoting their functional integration into restored motor networks, especially when compared to traditional, often passive or isolated rehabilitation methods (Butchbach and Scott, 2022). Whereas non-invasive neuromodulation techniques and motor rehabilitation both seek to enhance functional recovery, they function via distinct underlying mechanisms that may offer complementary pathways (Bonanno et al., 2022; Ding et al., 2022). The potential synergy lies in combining the precise neuromodulation to prime the SMA and related networks with motor rehabilitation to train the optimized circuits, potentially yielding greater functional gains than either approach alone (Bonanno et al., 2022; Ding et al., 2022). This may provide a theoretical basis for employing them individually or together in subacute stroke rehabilitation, given that neural remodeling during this phase calls for interventions that can both actively induce and meticulously guide the process.

In recent years, there has been increasing evidence supporting the effectiveness of non-invasive neuromodulation and motor rehabilitation interventions (Veldema and Gharabaghi, 2022; Badran et al., 2023; Liu et al., 2025), but their comparative efficacy and underlying mechanisms in SMA functional remodeling remain unclear, especially in subacute stroke. Existing evidence largely comes from small-sample studies or focuses on single interventions for stroke, lacking comparative studies (McClure et al., 2016; Liu et al., 2025). Furthermore, current reviews often mix acute and chronic stroke patients, failing to focus solely on the subacute phase, which limits the potential for optimizing rehabilitation protocols for this critical period. Therefore, there is an urgent need to explore the comparative efficacy and mechanisms of non-invasive neuromodulation and motor rehabilitation on SMA functional remodeling in subacute stroke patients.

Thus, this narrative review aims to synthesize existing literature, compare the efficacy of non-invasive neuromodulation and motor rehabilitation on functional reorganization of the SMA in subacute stroke, and explore their potential neural mechanisms. By integrating evidence, this review elucidates the synergistic or antagonistic effects of these interventions, fills current evidence gaps, and provides a theoretical basis for future research and clinical practice.

## 2 Main body

### 2.1 The role of the SMA in motor function reorganization in subacute stroke

#### 2.1.1 Anatomical and functional characteristics of the SMA

The SMA, situated medially within the frontal lobe and immediately anterior to the medial aspect of the precentral gyrus, constitutes an integral component of the human motor system, and its anatomical organization is characterized by close relationships with surrounding cortical and subcortical regions, most notably encompassing extensive connectivity with key motor-related structures such as the prefrontal cortex, M1, basal ganglia, and thalamus (Vergani et al., 2014; Dali et al., 2022; Zheng et al., 2025). The SMA can be further subdivided into two distinct subregions, namely SMA proper and pre-SMA, which exhibit differential functional involvement in motor execution and motor planning, respectively (Alario et al., 2006). Anatomical studies have shown that the SMA establishes close connections with the parietal lobe, basal ganglia, and other frontal motor areas through fiber pathways, such as the superior longitudinal fasciculus (SLF), frontal aslant tract (FAT), and fronto-caudate tract (FCT) (Komaitis et al., 2021; Vergani et al., 2021). These pathways provide the anatomical basis for the integration and transmission of motor information (Komaitis et al., 2021; Vergani et al., 2021). Meanwhile, the anatomical organization of the SMA enables it to receive inputs from sensory, cognitive, and motor systems, coordinating and integrating diverse motor-related signals to serve as a pivotal hub in the execution of complex motor behaviors (Catani et al., 2012; Umesawa et al., 2020). At the functional level, the SMA primarily contributes to motor planning, generation and execution of sequential movements, bilateral motor coordination, and initiation of voluntary actions (Padoa-Schioppa et al., 2002; Tabari et al., 2024). Classical neurophysiological and functional imaging studies have demonstrated that the SMA becomes activated during the motor preparation phase, particularly when generating complex sequential movements or coordinating bilateral limb actions, where its activity markedly increases (Klein et al., 2022; Fortier-Lebel and Nakajima, 2025). Furthermore, the SMA is closely associated with higher-order motor cognitive processes, like motor imagery and action observation, facilitating the preconfiguration and optimization of motor programs through internal simulation even without overt movement (Al-Wasity et al., 2021; Klein et al., 2022). In clinical practice, SMA injury often leads to motor initiation disorder, motor sequence disorder, and even SMA syndrome, manifested as transient motor weakness and speech disorders, which further emphasizes its central role in motor control (Pinson et al., 2022). Notably, the SMA also contributes critically to motor learning and skill acquisition, where its functional connectivity (FC) with prefrontal circuits and the basal ganglia supports the consolidation and transfer of motor programs (Nakahara et al., 2001; Perez et al., 2007; Censor et al., 2014).

After a stroke, the SMA is essential for motor recovery via compensatory strategies (Karbe et al., 1998). Studies have shown that when the M1 or associated motor pathways are impaired, the SMA can partially assume the functions of damaged regions by enhancing FC with contralateral motor areas and related brain regions, thereby facilitating the restoration of motor ability (Liu et al., 2019; Li et al., 2021). This compensatory process involves not only remodeling of structural connectivity but also reorganization of functional networks, such as strengthened interactions between the SMA and sensorimotor or attention networks, which contribute to the coordination of residual motor functions and improve rehabilitation outcomes (Sharp et al., 2011; Abidi et al., 2020; Parsons et al., 2023). Moreover, the plasticity of the SMA makes it a pivotal target for non-invasive neuromodulation like tDCS and TMS, as well as motor rehabilitation interventions, as modulating its activity facilitates motor function reconstruction (Grados et al., 2018; Kahl et al., 2021; Chen et al., 2023). Current multimodal neuroimaging and behavioral studies confirm that both the structural integrity and functional activity of the SMA are closely associated with motor recovery in stroke patients (Du et al., 2018; Quandt et al., 2019; Liu et al., 2020), suggesting that future rehabilitation strategies should emphasize targeted neuromodulation of the SMA and its associated networks to enhance therapeutic efficacy.

**Figure 1:**
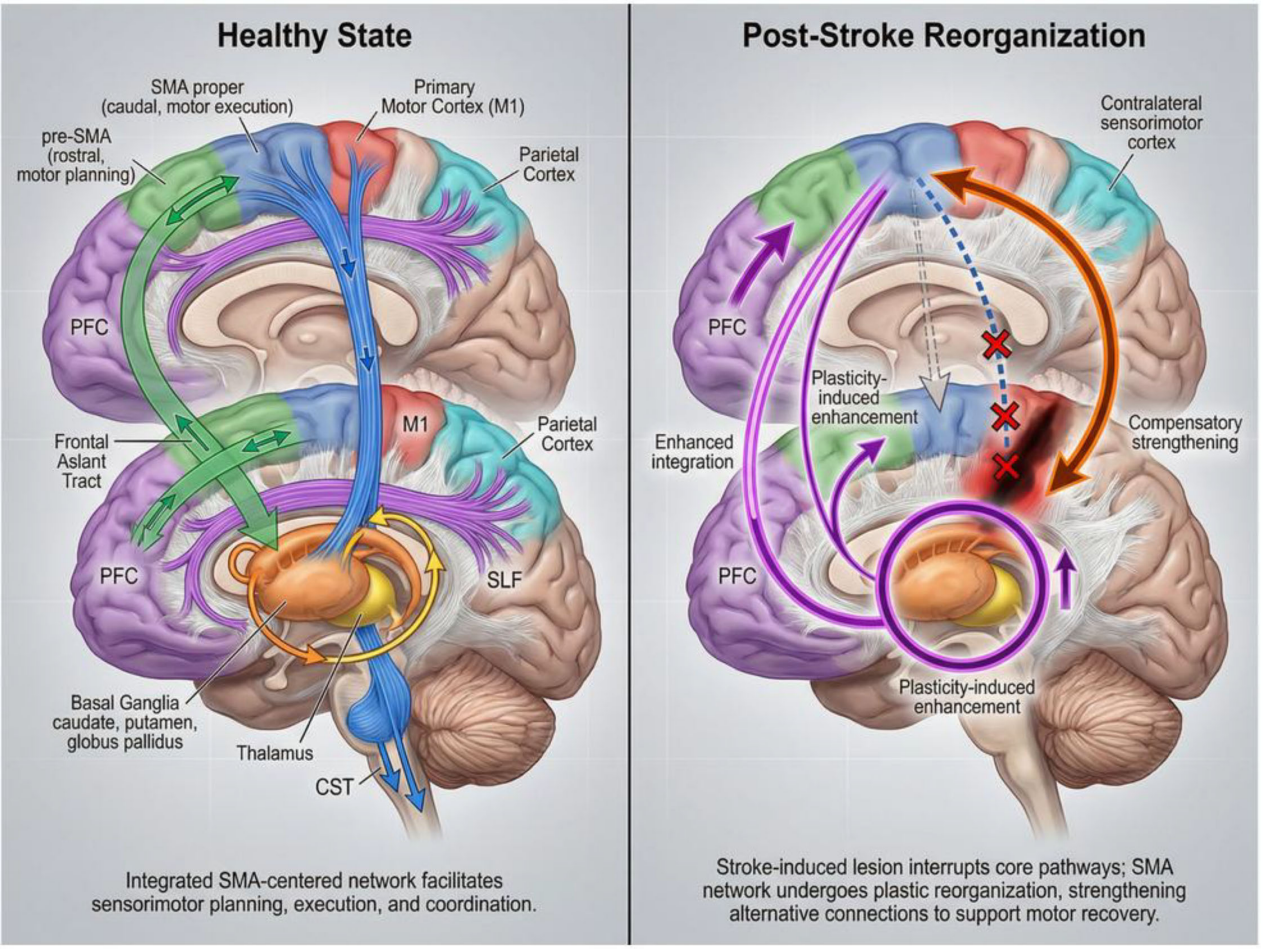
Anatomical network of the SMA and its remodeling after stroke.

In summary, as a key motor hub within the medial frontal lobe, the SMA not only undertakes essential functions in motor planning and coordination under physiological conditions, but also emerges as a critical node for functional motor recovery after neurological injuries such as stroke, owing to its remarkable plasticity and compensatory capacity. A deeper understanding of the anatomical architecture and functional properties of the SMA holds significant implications for refining neuromodulation and motor rehabilitation strategies, thereby improving motor outcomes in patients with subacute stroke. The anatomical basis of the SMA and its pattern of network reorganization after stroke are summarized in **Figure 1**.

#### 2.1.2 The function of the SMA in post-stroke neural network reorganization

Following stroke, the SMA serves as a pivotal hub in neural network reorganization, demonstrating particularly robust functional integration with regions, such as M1, basal ganglia, and thalamus (Westlake et al., 2012; Hannanu et al., 2017). Neuroimaging evidence from functional magnetic resonance imaging (fMRI) and electroencephalography (EEG) studies consistently indicates that FC between the SMA and M1 is crucial for motor recovery processes (Larsen et al., 2018; He et al., 2025). A systematic review indicates that frequent changes in FC in M1-SMA are closely related to improvements in motor function (Ismail et al., 2024), with this relationship observed during the subacute phase after stroke (Larsen et al., 2018; van Assche et al., 2021), suggesting that the SMA contributes not only to motor intention generation but also supports the reconstruction of motor output through its coupling with M1. Furthermore, enhanced connectivity between the SMA and core motor control regions, like the basal ganglia and thalamus, facilitates the reintegration and coordinated execution of motor programs (Liu et al., 2023), providing a structural basis for neuroplasticity in post-stroke patients.

During stroke rehabilitation interventions, the SMA exhibits markedly enhanced activity during both active and passive motor training paradigms (Ertelt et al., 2007; Xia et al., 2022). For instance, robot-assisted gait training enhances μ-band power in both the SMA and sensorimotor cortex (S1) post-intervention, with these neurophysiological changes correlating with improved lower limb muscle activation and gait function (Shin et al., 2023). Similarly, emerging technologies, such as virtual reality-based rehabilitation, have been demonstrated to strengthen FC between the SMA and other key nodes within the motor network, while promoting co-activation across motor and cognitive networks (Feitosa et al., 2022; He et al., 2025). These convergent findings substantiate the SMA’s role not merely as a motor execution center, but as a pivotal hub for neural reorganization and functional restoration post-stroke. It is noteworthy that there is a close positive correlation between increased SMA activity and the degree of motor function recovery (Hannanu et al., 2017). Multiple studies have found that the higher the SMA activation level, the more significant the improvement in the patient’s motor ability, especially in the subacute phase (Saur et al., 2006; van Assche et al., 2021; Ma et al., 2025). For example, both subacute and chronic stroke patients showed significant upregulation of SMA activity after receiving different types of exercise rehabilitation, accompanied by improved motor function, but this was more pronounced in the subacute phase (Nasrallah et al., 2021; Shin et al., 2022). During the recovery of motor function in the upper and lower limbs, the SMA is synergistically activated with networks in areas such as M1, S1, the parietal lobe, and the cerebellum, further promoting the comprehensive recovery of motor function (Catalogna et al., 2023; Hanakawa et al., 2023). Based on these findings, it can be inferred that the SMA not only integrates motor commands but also provides a neural substrate for functional motor recovery through functional coupling with other key regions during post-stroke motor network reorganization. Moreover, the plasticity of the SMA extends beyond FC enhancements to encompass structural reorganization (Yuan et al., 2020). Longitudinal neuroimaging studies have demonstrated that increased grey matter volume in the SMA correlates significantly with motor recovery post-stroke, with these structural changes occurring in concert with remodeling in regions, such as the cerebellum and thalamus (Hanakawa et al., 2023). These findings indicate that neural reorganization following stroke involves both functional adaptations and anatomical restructuring within the SMA, providing dual mechanisms supporting motor recovery. Collectively, through its functional and structural integration with M1, basal ganglia, and thalamic regions (Pollok et al., 2009), the SMA establishes a robust neural foundation for motor function restoration in stroke patients.

Collectively, the SMA serves a bridging and integrative function in post-stroke neural network reorganization, with its enhanced FC to motor-related regions representing a key indicator of motor recovery (Westlake et al., 2012). Furthermore, increased SMA activity not only reflects neural network plasticity but also directly correlates with improved motor function (Du et al., 2025), particularly during the subacute rehabilitation phase. Consequently, targeted modulation of the SMA and its network connections emerge as a promising therapeutic strategy for future stroke motor rehabilitation interventions.

#### 2.1.3 Imaging evidence supports the SMA functional remodeling

fMRI has provided key evidence in studies of functional remodeling of the SMA after stroke. Studies demonstrate that stroke patients exhibit significantly enhanced activation in the SMA following neuromodulation or motor rehabilitation interventions, with this change closely correlating with motor functional recovery (Grefkes et al., 2010; Manganotti et al., 2010). For instance, non-invasive brain stimulation techniques can modulate cortical excitability and induce remote functional changes within motor networks, thereby promoting upper limb motor improvement (Chieffo et al., 2016). These neuroimaging findings not only reveal the central role of the SMA in motor recovery but also suggest that enhanced SMA activation may represent a marker of neuroplastic reorganization. When combined with motor training, patients demonstrate strengthened functional engagement of the SMA and associated motor networks on fMRI, further supporting its involvement in functional restoration (Papoutsi et al., 2018). Notably, studies have observed that lateralization patterns of SMA activation may dynamically shift throughout rehabilitation, reflecting the ongoing nature of functional reorganization in the brain (Catalogna et al., 2023). Structural MRI and diffusion tensor imaging (DTI) also provide direct evidence for plasticity changes in SMA-related fiber bundles. Following a stroke, DTI can detect alterations in the integrity and connectivity of white matter fibers between the SMA and other motor-related brain regions (Han et al., 2019). For example, during rehabilitation, increased fractional anisotropy (FA) values in SMA-associated fiber tracts indicate repair and remodeling of structural fibers (Ruddy et al., 2017). Additionally, animal studies have demonstrated that neuromodulation at specific frequencies combined with motor training enhances plasticity in SMA-related fiber tracts and improves motor performance (Pekala et al., 2018), thereby offering a theoretical foundation for clinical intervention strategies.

Multimodal neuroimaging approaches have deepened our mechanistic understanding of functional reorganization within the SMA. Integrated analysis of functional and structural data reveals that enhanced SMA activation is frequently accompanied by structural repair of its associated fiber tracts, with these parallel changes acting synergistically to support motor recovery (Liu et al., 2020). Notably, combined fMRI and DTI assessments during the subacute phase of stroke can sensitively capture these plastic changes within the SMA and its associated networks, which may help predict rehabilitation outcomes and guide personalized therapeutic strategies (Chen et al., 2018). Critically, the consistent co-occurrence of SMA functional augmentation and structural reinforcement suggests that functional and structural remodeling are likely interdependent and co-evolve during recovery (Kulkarni et al., 2021). Neuroimaging evidence has further revealed considerable interindividual variability in the functional reorganization of the SMA (Konrad et al., 2002; Jiménez de la Peña et al., 2024). Significant differences exist among patients in SMA activation patterns and fiber bundle remodeling amplitude (Fujimoto et al., 2014; Quandt et al., 2019), which may be related to stroke location, degree of injury, and type of rehabilitation intervention. Therefore, image-based individualized assessment is critical for optimizing rehabilitation strategies. Future advances in high-resolution neuroimaging techniques will enable more precise characterization of the spatiotemporal dynamics of SMA plasticity, thereby providing a robust theoretical framework for precision rehabilitation.

**Figure 2:**
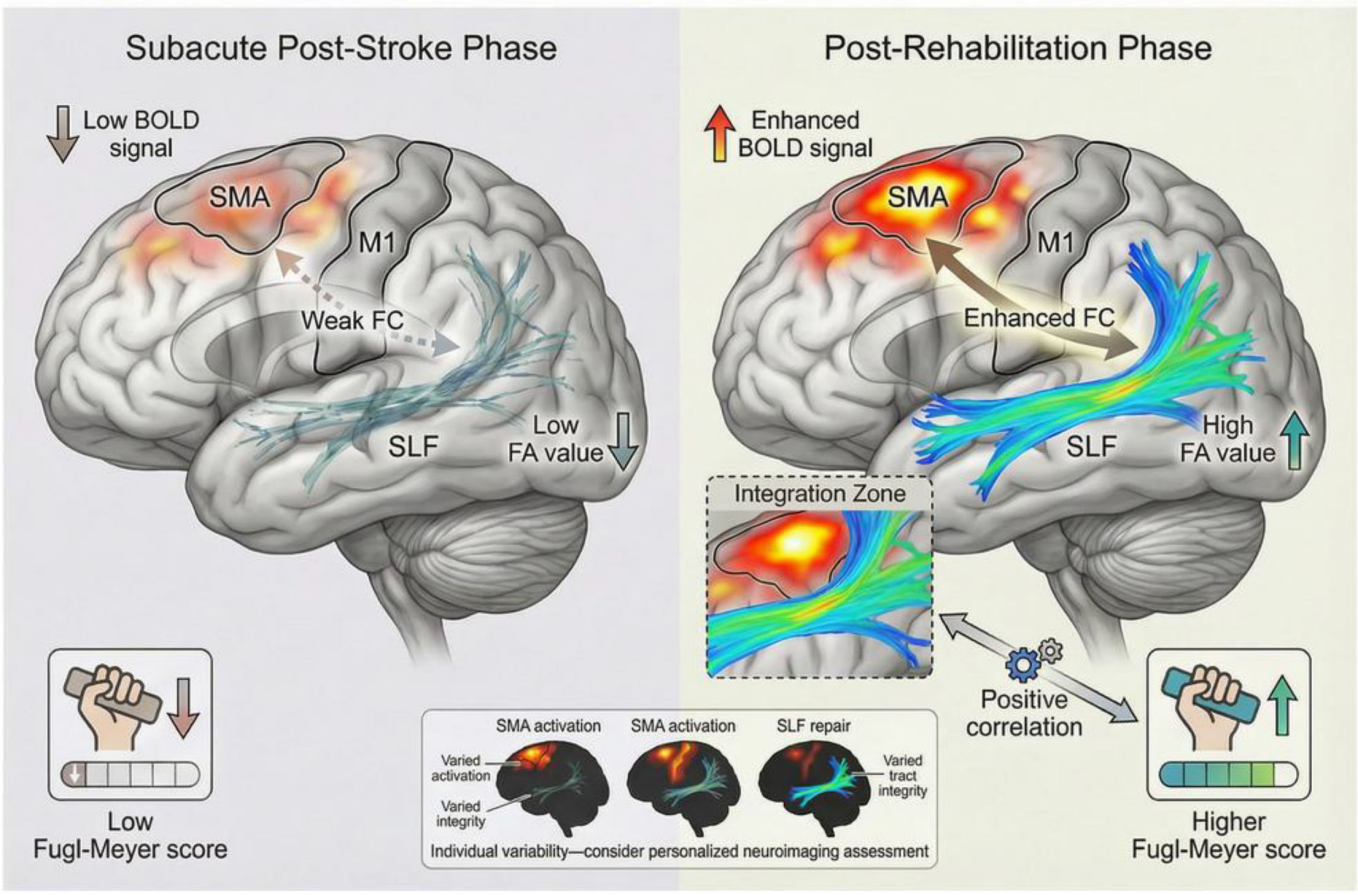
Summary of multimodal neuroimaging evidence supporting SMA functional remodeling.

In summary, imaging studies have provided solid evidence for the role of SMA in post-stroke functional remodeling and motor rehabilitation. These findings not only enrich the theory of stroke neuroplasticity but also provide important references for the assessment and optimization of clinical rehabilitation interventions. **Figure 2** summarizes the multimodal neuroimaging evidence supporting SMA functional remodeling.

### 2.2 Effects of non-invasive neuromodulation on the SMA functional reorganization

#### 2.2.1 Mechanism of rTMS on the SMA

rTMS is a non-invasive neuromodulation technique that can promote the functional reorganization of neural networks by modulating cortical excitability in the SMA (Lv et al., 2023). The efficacy and neural mechanisms of rTMS are highly dependent on precise stimulation parameters, including frequency, intensity, duration, and target location within the SMA. For frequency, both high-frequency (e.g., 5 Hz, 10 Hz) and low-frequency (e.g., 1 Hz) protocols are employed, with distinct neuromodulatory effects (Hamada et al., 2008; Shirota et al., 2013). High-frequency rTMS is often used to activate the affected or dysfunctional SMA, with a standard protocol consisting of a 10-second stimulation train (50–100 pulses) with a 20–30 second interval between trains. Low-frequency rTMS, on the other hand, is used to suppress overexcitation of the contralateral (healthy) SMA or M1, and often employs a continuous stimulation pattern (Fujimoto et al., 2014; Lv et al., 2023). The stimulation intensity is typically expressed as a percentage of the individual’s resting motor threshold (RMT). For rTMS studies targeting the SMA, the intensity often falls within the range of 80% to 110% of the RMT (Hamada et al., 2008; Shirota et al., 2013). To mitigate the risk of inducing seizures, the intensity for high-frequency stimulation is commonly set around 90% of the RMT, whereas low-frequency stimulation may utilize a slightly higher intensity (e.g., 100-110% of the RMT) to achieve effective inhibition (Hamada et al., 2008; Shirota et al., 2013; Kahl et al., 2021). Regarding the treatment course, the treatment cycle varies considerably in clinical studies, ranging from once a day for 1–2 weeks to several times a week for several weeks (Hamada et al., 2008; Shirota et al., 2013; Kahl et al., 2021). Stimulation is precisely anatomically targeted, typically focusing on the anterior SMA (more associated with motor learning and cognitive control) or the SMA proper (more related to motor execution and preparation), with targeting accuracy often ensured through individualized structural MRI or navigation based on the international 10−20 EEG system, for example by aiming for a midline position 1−2 cm anterior to the Fz point (Hamada et al., 2008; Shirota et al., 2013; Flamez et al., 2021; Kahl et al., 2021).

Multiple studies support the idea that rTMS stimulation of the SMA can significantly improve motor function in patients with various neurological disorders. For example, in Parkinson’s disease patients, weekly application of 5 Hz high-frequency rTMS over the left SMA for 8 weeks significantly improved UPDRS scores and motor performance, and fMRI showed enhanced activation in motor network-related regions, such as the right precentral gyrus, superior frontal gyrus, middle frontal gyrus, thalamus, and cerebellum (Bhat et al., 2023). Similarly, rTMS enhances FC within the motor network of stroke patients, particularly strengthening the connectivity between the SMA and bilateral M1 as well as premotor areas (PMA), which is closely correlated with motor recovery (Guo et al., 2021). It is worth noting that the regulation mechanism of SMA differs among rTMS at different frequencies. High-frequency (≥5 Hz) rTMS directly activates the impaired SMA, promotes neuronal regeneration and the reorganization of FC, and enhances motor recovery. A randomized controlled trial (Zhao et al., 2024) in patients with subacute stroke provides a specific parameter paradigm in the intervention group: applying 10 Hz high-frequency rTMS over the ipsilesional SMA (localized to FC1 or FC2 according to the 10–10 international EEG system), with a stimulation intensity at 100% of the individual’s RMT, each train lasting 2.5 seconds with an inter-train interval of 10 seconds, resulting in a total treatment duration of 20 minutes per session containing 2400 pulses, administered once daily, 5 days a week for 4 weeks. This protocol demonstrated superior improvements in both upper and lower limb Fugl-Meyer assessment scores and berg balance scale scores compared to the control group. In contrast, low-frequency (≤1 Hz) rTMS can suppress excessive inhibitory responses in the contralateral SMA, restore functional balance between bilateral motor regions, and reduce inhibitory imbalance following stroke. Studies have shown that applying 1 Hz rTMS over the contralesional M1 in stroke patients reduces inhibitory drive from the contralesional M1 (110% RMT, continuous stimulation for 20 minutes delivering 1200 pulses per session, daily for 10 days) to the ipsilesional M1 and decreases resting-state FC from the contralesional M1 to the ipsilesional PMA and SMA, thereby promoting interhemispheric balance and motor recovery (Sánchez-Cuesta et al., 2021; Scho et al., 2024). Besides, fMRI revealed enhanced FC between the SMA and the ipsilateral cerebellum, a change that was positively correlated with improvements in balance function (Zhao et al., 2024). Additionally, rTMS stimulation of SMA can further promote the plasticity and reorganization of motor networks by regulating cerebral blood flow and white matter microstructure in local brain regions (Jin et al., 2022). In stroke patients, rTMS combined with bilateral upper limb training can synergistically enhance the information inflow and dominant information flow in the SMA and related motor areas, suggesting its synergistic effect in motor function reconstruction (Li et al., 2025). It is worth noting that rTMS regulates SMA not only by changes in local cortical excitability, but also by network remodeling in distant brain regions (Matsunaga et al., 2005). For instance, fMRI combined with multivariate pattern analysis has revealed that rTMS elicits functional enhancement in distributed regions of the motor network, such as the cerebellum and thalamus, which underpins the neural basis for the restoration of motor function (Bhat et al., 2023). The SMA acts as a flexible hub, supporting motor learning by facilitating regional activation and coordinating neural coupling across brain regions, particularly between hemispheres (Chen et al., 2025). While high-frequency rTMS is applied, the FC between the SMA and the ipsilateral M1, as well as the contralateral PMA, is significantly augmented, which correlates strongly with enhancements in motor function, suggesting that high-frequency rTMS may be more conducive to promoting the reorganization of the motor network centered on the SMA (Guo et al., 2021).

Synthesis of both basic and clinical evidence indicates that rTMS exerts a beneficial role in the rehabilitation of motor dysfunction by modulating cortical excitability within the SMA and promoting functional reorganization of key motor networks, particularly the SMA-motor cortex circuitry. The efficacy and direction of these effects are critically dependent on precise stimulation parameters, including the targeted SMA subregion (e.g., pre-SMA *vs* SMA-proper located via anatomical MRI or neuronavigation), frequency (e.g., inhibitory 1 Hz *vs* facilitatory 5–10 Hz), intensity (typically 80%-110% RMT), and treatment regimen (e.g., total pulses per session and number of sessions). Recent research further confirms that rTMS can promote motor recovery after stroke by rebalancing cortical excitability and reducing maladaptive connectivity patterns (Yang et al., 2026). Although different frequencies and stimulation parameters exhibit distinct regulatory profiles on the SMA and its associated networks, the most substantial evidence supports the use of high-frequency rTMS to enhance FC between the SMA and motor cortical regions, thereby facilitating improvements in motor performance. Future integration of individualized neuroimaging assessments is expected to refine rTMS parameters and advance precision neuromodulation strategies for motor recovery.

In summary, the mechanisms of action underlying rTMS targeting the SMA operate primarily through the modulation of cortical excitability and functional reorganization of motor networks. Low-frequency rTMS focuses on inhibiting contralateral hyperactivity to restore interhemispheric balance, while high-frequency rTMS directly activates and enhances the functional connection between the SMA and the motor cortex, thereby jointly promoting the recovery of motor function. Setting the parameters is crucial to ensuring its clinical efficacy and scientific value. This evidence supports a novel neuromodulatory strategy for the rehabilitation of motor dysfunction in patients with subacute stroke.

#### 2.2.2 Modulatory effects of tDCS on the SMA

tDCS is a non-invasive neuromodulation technique that can modulate neuronal excitability and synaptic plasticity by altering membrane potentials in targeted cortical regions (Qi et al., 2024). tDCS interventions over the SMA have demonstrated unique therapeutic potential for motor function rehabilitation and neural network reorganization (Hasui et al., 2022). In clinical and research applications targeting the SMA, typical tDCS parameters include a low-intensity direct current of 1–2 mA, applied for 20–30 minutes per session, using electrodes sized 25–35 cm² (Kirimoto et al., 2011; Soltani et al., 2026). The anode (for excitatory stimulation) or cathode (for inhibitory stimulation) is precisely positioned over the SMA based on the international 10–20 EEG system (often at FCz coordinates) or through neuronavigation guided by individual MRI to target specific SMA subregions (e.g., pre-SMA or SMA-proper), depending on the rehabilitation goals (Kirimoto et al., 2011; Shibasaki, 2012; Soltani et al., 2026). By delivering low-intensity direct currents to the SMA, tDCS induces mild neuronal depolarization (anodal stimulation) or hyperpolarization (cathodal stimulation), thereby modulating local neural circuit activity and promoting plasticity through both presynaptic and postsynaptic mechanisms (Meng et al., 2024). These neurophysiological changes establish a foundation for motor recovery, particularly during early-stage neural network reconstruction following stroke.

When the anodic tDCS stimulates the SMA, it can increase the neural excitability of the region, thereby promoting the activation and reorganization of motor-related neural circuits (Kirimoto et al., 2011; Coelho et al., 2025). Studies in healthy individuals have shown that anodal tDCS over SMA (e.g., 1 mA for 10 min) can improve performance on simple and choice reaction time tasks, suggesting enhanced motor planning and execution (Carlsen et al., 2015; Hupfeld et al., 2017). In patient populations, anodal stimulation has demonstrated therapeutic potential. For instance, in Parkinson’s disease, applying anodal tDCS (1 mA, 10 min) over the SMA significantly improved upper limb movement kinematics, such as reducing time to peak displacement and movement time (Sadler et al., 2021). In stroke rehabilitation, combining anodal tDCS (1 mA, 20 min, anode at Cz, cathode at inion) with body weight-supported treadmill training significantly improved gait speed and functional walking ability compared to sham stimulation (Manji et al., 2018). Furthermore, anodal tDCS over SMA (2 mA, 15 min) has been shown to enhance anticipatory postural adjustments and improve postural sway during rapid arm movements in older adults (Nomura and Kirimoto, 2018). These effects may involve not only enhanced regional activation of the SMA itself but also the modulation of broader motor control networks through cortico-cortical or cortico-spinal pathways, and such network-level regulation is considered a pivotal mechanism underlying tDCS-facilitated motor rehabilitation (Hirabayashi et al., 2020).

In contrast, cathodal tDCS applied to the SMA reduces regional neuronal excitability, thereby suppressing aberrant neural activity (Carlsen et al., 2015). This approach has been explored in conditions characterized by presumed SMA hyperexcitability. For example, in Tourette syndrome, cathodal tDCS applied over the bilateral SMA (1 mA, 20 min twice daily for 5 days) led to a significant reduction in motor tic severity and premonitory urge intensity (Mahjoub et al., 2025). A single session of cathodal tDCS over SMA also showed potential in reducing tic impairment scores (Dyke et al., 2019). In obsessive-compulsive disorder (OCD), cathodal tDCS targeting circuits involving the pre-SMA (e.g., cathode over pre-SMA with anode over orbitofrontal cortex) has been investigated as a treatment for resistant symptoms, with some protocols showing symptom reduction (Chu et al., 2025). Additionally, studies have demonstrated that cathodal tDCS applied to the SMA enhances motor inhibition and executive control (Hsu et al., 2011), thereby introducing innovative intervention strategies for patients with motor abnormalities or impulse control disorders after subacute stroke. The mechanism may involve increased inhibitory control, as suggested by studies where cathodal tDCS over SMA slowed reaction time and decreased the incidence of premature movement release (Carlsen et al., 2015).

Consequently, the choice between anodal and cathodal stimulation, as well as the precise targeting of SMA versus pre-SMA, must be individualized based on the patient’s pathophysiology and rehabilitation goals. Furthermore, the efficacy of tDCS in modulating SMA function is influenced by multiple factors, including stimulation parameters (e.g., current intensity, duration, electrode size and placement), interindividual neuroanatomical variations, and integration strategies with rehabilitation training (Lee and Yoo, 2024). Specifically, the selection of stimulation parameters (e.g., 1 mA *vs* 2 mA; 20 *vs* 30 minutes) and target location (pre-SMA for cognitive-motor integration *vs* SMA-proper for pure motor execution) should be tailored to the specific pathophysiological state and recovery stage of subacute stroke patients. Several studies indicate that repeated, periodic tDCS sessions (e.g., daily sessions for 2–4 weeks) produce more sustained neuroplastic effects compared to single applications, while synergistic combination with motor training further enhances clinical benefits (Chow et al., 2022; Longo et al., 2022). Emerging evidence suggests that multi-target stimulation engaging both the SMA and functionally connected networks, like combined with M1, may surpass isolated stimulation in promoting comprehensive motor recovery (Chen et al., 2019; Guo et al., 2022).

Collectively, tDCS modulates membrane potentials in the SMA, thereby promoting plasticity within motor-related neural networks or suppressing pathological activity. Anodal stimulation facilitates the enhancement of motor function, whereas cathodal stimulation is suitable for inhibiting aberrant neural excitability. In the future, combining individualized parameter optimization and multimodal rehabilitation strategies is expected to further enhance the application value of tDCS in subacute stroke rehabilitation.

#### 2.2.3 Potential of emerging non-invasive neuromodulation techniques on the SMA

While rTMS and tDCS are the most extensively studied non-invasive neuromodulation techniques in stroke rehabilitation, emerging technologies such as transcranial alternating current stimulation (tACS) and transcranial focused ultrasound (tFUS) neuromodulation are rapidly advancing the field, offering novel mechanisms and potentially superior spatial precision for targeting deep brain structures like the SMA. Incorporating these techniques into the therapeutic arsenal may provide more individualized and effective strategies for promoting functional reorganization in subacute stroke.

tACS is fundamentally different from tDCS in its mechanism, as it applies sinusoidal oscillatory currents on the scalp to primarily entrain or resonate endogenous neuronal oscillations, rather than inducing a sustained shift in resting membrane potential (Zaehle et al., 2010). By delivering current at specific frequencies (e.g., alpha: 8–12 Hz, beta: 13–30 Hz), tACS can potentially enhance or disrupt pathological oscillatory rhythms within and between brain regions (Zaehle et al., 2010). This frequency-specific approach is particularly relevant for the SMA, a key node in the sensorimotor network where aberrant beta-band oscillations are often implicated in post-stroke motor deficits (Lai et al., 2025). Theoretically, applying beta-tACS over the SMA could enhance physiological beta rhythms associated with motor maintenance and preparation, thereby improving FC between the SMA and the M1 and facilitating motor planning processes (Miyaguchi et al., 2020; Lai et al., 2025). A case report provides preliminary clinical evidence for this approach (Lai et al., 2025). Researchers performed a two-week, once-daily multi-target 40Hz tACS on a patient with post-stroke cognitive impairment, simultaneously stimulating the dorsolateral prefrontal cortex, M1, and SMA, in conjunction with intensive cognitive rehabilitation (Lai et al., 2025). This intervention improved the patient’s cognitive and motor performance, with neurophysiological (TMS, EEG) and neuroimaging (MRI) evidence suggesting enhanced cortical excitability and network synchronization (Lai et al., 2025). This reveals a new strategy of using frequency-specific, multi-node stimulation to modulate the SMA as a key hub within distributed motor-cognitive networks (Lai et al., 2025). Besides, preliminary clinical evidence supporting this strategy comes from a pilot randomized controlled trial involving patients with subacute stroke (Yamashita et al., 2025). This study explored the feasibility and effects of combining tDCS targeted at the SMA with gait-synchronized rhythmic tACS applied to the M1during treadmill walking training. The pilot findings demonstrated that the combined intervention, administered over 3 weeks for a total of 15 sessions, was well-tolerated and feasible. It significantly reduced gait temporal variability on the paretic side and concurrently improved balance function in the participants. This provides direct preliminary efficacy evidence and a practical protocol reference for applying tACS synchronized with behavioral training (e.g., walking) in a multi-target modality (e.g., simultaneously modulating the SMA and M1) for subacute stroke rehabilitation. Although direct clinical trials of SMA-targeted tACS in stroke populations are still in their early stages, and the optimization of its key parameters (e.g., frequency, intensity, phase) requires further exploration, its oscillation-based, network-level regulatory capacity offers a promising new direction for achieving highly individualized and mechanistically-defined neural functional reorganization.

tFUS neuromodulation represents a paradigm shift in non-invasive brain stimulation, utilizing focused acoustic pressure waves rather than electromagnetic fields to influence neural activity, and its unique advantages of millimeter-level spatial precision and the capacity for non-invasive targeting of deep brain structures offer distinct potential for the precise modulation of regions such as the SMA (Zhang et al., 2021; Li et al., 2024; Jiang et al., 2025). The efficacy of tFUS depends critically on key sonication parameters, including ultrasound frequency (typically 0.25–0.5 MHz), intensity (e.g., spatial peak pulse average intensity, often 1–30 W/cm²), pulse repetition frequency, stimulation duration per session, and neuronavigation-guided targeting (Zhang et al., 2021; Li et al., 2024; Jiang et al., 2025). Theoretically, during the critical window of neuroplasticity in the subacute phase post-stroke, low-intensity focused ultrasound could instantaneously modulate neuronal excitability via mechanical and thermal effects, thereby precisely upregulating excitability in the perilesional SMA or suppressing maladaptive hyperactivity within connected networks (Li et al., 2023; Qi et al., 2024; Jiang et al., 2025). Beyond this direct neuromodulation, its more revolutionary potential lies in ultrasound-mediated neuro-immunomodulation and targeted drug delivery. Innovative ultrasound-responsive biomaterials, such as hydrogels, have been shown to enable the spatiotemporal controlled release of bioactive factors, such as resveratrol, thereby precisely suppressing excessive inflammatory peaks following tissue injury and creating a favorable molecular microenvironment for repair (Han et al., 2023). This principle holds great promise for regulating secondary neuroinflammation following stroke. Concurrently, ultrasound-mediated transient opening of the blood-brain barrier (BBB) ​​opens a breakthrough pathway for targeted delivery of neuroprotective or regenerative drugs, enabling precise intervention in the SMA and its networks (Jiang et al., 2025). Further integration with tissue engineering, such as employing piezoelectric nanomaterials to convert acoustic energy into localized biomimetic electrical signals, enables multi-modal regulation of the neural microenvironment (Han et al., 2025). Collectively, these strategies may aim to promote SMA functional reorganization by precisely modulating the associated inflammatory, immune, and plasticity-related neural milieu. In contrast to rTMS and tDCS, which primarily modulate electrophysiological balance, tFUS provides a broader toolkit encompassing mechanical neuromodulation, immunomodulation, and targeted delivery (Miyaguchi et al., 2020; Lai et al., 2025; Yamashita et al., 2025). Although clinical research on tFUS specifically targeting the SMA in subacute stroke remains nascent, its interdisciplinary progress underscores immense potential for fostering functional recovery through highly precise, programmable interventions. Future efforts should focus on optimizing tFUS parameters for SMA network modulation, exploring its synergistic integration with motor rehabilitation, and investigating combined strategies with rTMS/tDCS to develop stratified, precision neuromodulation therapies for targeted functional improvement in subacute stroke patients.

In summary, alongside rTMS and tDCS, techniques such as tFUS and tACS provide complementary and innovative approaches for modulating the SMA. tFUS enables deep targeting with unparalleled spatial precision, while tACS offers frequency-specific network entrainment. Their successful integration into subacute stroke rehabilitation paradigms hinges on further translational research to optimize parameters, validate their safety and efficacy in patient populations, and delineate their synergistic value when combined with behavioral motor training. Pursuing these avenues will undoubtedly enrich the toolkit for promoting SMA functional reorganization and improving rehabilitative outcomes.

#### 2.2.4 Neuroimaging and neurophysiological evidence

Neuroimaging and neurophysiological techniques have provided important evidence for revealing the role of non-invasive neuromodulation and motor rehabilitation in the functional remodeling of the SMA in patients with subacute stroke. fMRI studies have shown that interventions using neuromodulation techniques, such as rTMS and tDCS, significantly enhance the activation of SMA and its related motor networks and improve FC between brain regions during motor execution and imagination tasks (Yoo et al., 2008; Guo et al., 2021). For instance, one study demonstrated that a 4-week course of rTMS targeting the ipsilesional SMA in patients within 12 weeks post-stroke specifically enhanced FC between the ipsilesional SMA and the ipsilateral cerebellum (Zhao et al., 2024). This enhancement was positively correlated with improvements in balance function (r=0.530, p=0.029). Further investigation revealed that the similar intervention also strengthened resting-state FC between the contralateral cerebellar dentate nucleus and the ipsilateral ventral PMA (Zhao et al., 2025). The degree of this enhancement showed a strong positive correlation with balance recovery (r=0.637, p=0.001). Collectively, these findings suggest that neuromodulation targeting the SMA can concurrently modulate activity in multiple anatomically and functionally related brain regions, including the premotor cortex and cerebellum. Importantly, the increased FC within these specific circuits is directly associated with the improvement of clinical motor functions, particularly balance. Besides, during non-invasive neuromodulation combined with hand grasping movement tasks in subacute stroke patients, notable alterations in FC have been observed among the premotor cortex, M1, S1, and networks involving frontal, parietal, and occipital lobes across both low- and high-frequency bands (Lin et al., 2023). Compared to chronic patients, those in the subacute phase demonstrate higher global network transmission efficiency yet weaker nodal centrality, suggesting active dynamic remodeling within motor-related networks during this stage (Lin et al., 2023). These alterations establish a foundation for understanding the patterns of cerebral network reorganization and mechanisms of spontaneous recovery across different post-stroke phases. At the neurophysiological level, metrics such as event-related potentials (ERP), motor evoked potentials (MEP), and somatosensory evoked potentials (SEP) are widely utilized to assess functional integrity of the SMA and associated motor networks. Studies indicate that non-invasive neuromodulation enhances electrophysiological activity in central motor regions, manifested through increased event-related desynchronization (ERD) over centro‐motor areas and flattened EEG power spectral density, reflecting elevated motor cortical plasticity (Xu et al., 2025). Further FC analysis demonstrates that the interactions between the SMA and sensorimotor, language, and attention networks are significantly enhanced, which is particularly evident in stroke patients exhibiting substantial clinical improvement (Liu et al., 2019). Recent structural MRI evidence further supports this, demonstrating that rTMS targeting the SMA induces a significant increase in gray matter volume (GMV) in the posterior cerebellum and cerebellar vermis lobules. This structural change shows a moderately positive correlation with improvements in balance function (r=0.436, p=0.038) (Zhao et al., 2025). Individual variability in FC reorganization is observed across patients with distinct lesion profiles, with a critical window for neuroplastic remodeling occurring approximately 3 weeks post-intervention (Cao et al., 2025). Notably, alterations in neurophysiological signals such as ERP exhibit strong correlations with motor recovery, providing an objective basis for evaluating rehabilitation efficacy. Furthermore, the application of neuromodulation techniques such as tDCS in motor rehabilitation is corroborated by electrophysiological and neuroimaging evidence. The combination of tDCS with extended reality training enhances neural activity in the ipsilesional M1, improves electromyographic activity and joint mobility in paralyzed muscles, and significantly reduces the brain symmetry index (BSI), suggesting concurrent cerebral functional reorganization and motor recovery (Ruas et al., 2025). Further research indicated that following dual-channel tDCS targeting bilateral S1 combined with task-oriented sensorimotor training, improvements in the Fugl-Meyer assessment for upper extremity showed a strong positive correlation with the strength of FC between the right premotor cortex and the left SMA/left somatosensory association cortex (r=0.815, p<0.001) (Li et al., 2024). Additionally, fMRI studies demonstrate that integrated motor training expands the activation range of the SMA and associated networks, with some patients exhibiting synergistic bilateral M1 activation, indicating that plastic reorganization of the motor cortex contributes to functional restoration (Gould et al., 2021). Another study that applied anodal tDCS over the ipsilesional lower limb motor area combined with robotic gait training also confirmed that the intervention group exhibited significantly stronger FC between the left M1 and the right premotor cortex/SMA compared to the control group, alongside more pronounced improvements in lower limb motor function (Zhang et al., 2025). Collectively, these neuroimaging and neurophysiological findings substantiate that non-invasive neuromodulation combined with motor rehabilitation promotes functional reorganization of the SMA and its connected networks, establishing a biological foundation for developing individualized rehabilitation strategies. Representative studies detailing SMA-related neuroimaging changes and clinical functional improvements following interventions in subacute stroke are summarized in **Table 1**. While the evidence summarized above robustly supports the neuromodulatory effects of rTMS and tDCS on SMA networks, the neuroimaging and electrophysiological evidence base for emerging techniques like tACS and tFUS, particularly in the context of subacute stroke, is still being established. Preliminary research and case reports suggest that tACS can modulate network-level oscillatory synchrony, and tFUS may induce measurable, localized changes in neural activity and connectivity. These effects have been inferred from concurrent EEG or fMRI recordings in studies of other neurological conditions or in healthy participants. However, targeted and controlled studies specifically examining SMA reorganization in subacute stroke populations using these newer modalities are needed to translate their promising mechanistic potential into validated clinical evidence.

**Table 1:** Exemplary studies on SMA-related neuroimaging changes and clinical functional improvements following interventions in subacute stroke.

Studies	Design	Sample characteristics	Intervention details	Control	Key neuroimaging findings (SMA-related)	Clinical outcome measure	Reported correlation/association
Zhao et al. (2025)	RCT	N=69 SMA group: 23 M1 group: 23 CG: 23 Within 12 weeks post-stroke	Modality: rTMS targeting the affected SMA and M1 separately Localization: based on the 10–10 international EEG standard M1-midpoint of the line connecting CPz to C3 or C4, SMA-FC1 or FC2 Stimulation frequency: 10 Hz Intensity: 100% RMT Stimulation time: 2.5 s Interval time: 10 s Total time: 20 minutes Duration: once a day, 5 days a week, 4 weeks rTMS followed by regular rehabilitation incorporating motor training as CG	Sham rTMS: midpoint of Pz and C3/C4 Regular rehabilitation, 5 days a week, 4 weeks	FC analysis revealed significantly increased rsFC in the contralateral dentate nucleus and ventromedial premotor area of the affected side in the SMA group relative to the M1 group (p=0.0319) Structural MRI analysis demonstrated that while the M1 group showed a significant increase in GMV in the medial segment of the postcentral gyrus (p=0.02), the SMA group displayed significant GMV increases in the posterior cerebellum and vermal lobules (p=0.0428)	The SMA group showed significantly superior improvement in motor function, particularly in balance function, compared to the M1 group (p<0.05)	The enhancement of rsFC between the contralateral dentate nucleus and ventromedial premotor area of the affected side was strongly positively correlated with improvements in balance function (r=0.637, p=0.001) Increased GMV in the posterior cerebellum demonstrated a moderate positive correlation with improvement in balance function (r=0.436, p=0.038)
Zhao et al. (2024)	RCT	N=54 SMA group: 18 M1 group: 18 CG: 18 2-12 weeks post-stroke	Modality: rTMS targeting the affected SMA and M1 separately Localization: based on the 10–10 international EEG standard M1-midpoint of the line connecting CPz to C3 or C4, SMA-FC1 or FC2 Stimulation frequency: 10 Hz Intensity: 100% RMT Stimulation time: 2.5 s Interval time: 10 s Total time: 20 minutes Duration: once a day, 5 days a week, 4 weeks	Sham rTMS: midpoint of Pz and C3/C4	The SMA group demonstrated increased FC between the affected SMA and the ipsilateral cerebellum post-intervention compared to the M1 group and CG (p<0.05)	The SMA group demonstrated significantly greater improvements in balance function scores compared to both the M1 group (p=0.034) and the CG (p=0.012)	Within the SMA group, a positive correlation was observed between changes in FC linking the affected SMA and the ipsilateral cerebellum and improvements in balance function (r=0.530, p=0.029)
Li et al. (2024)	RCT	N=52 IG: 26 CG: 26 14–180 days post-stroke	Modality: dual-tDCS targeting the bilateral S1, combined with task-oriented sensorimotor training Localization: the optimal stimulation point for the M1 was located using TMS. The S1 stimulation point was located 2 cm posterior to the M1 point, parallel to the midline. The cathode was placed over the S1 contralateral to the lesion, and the anode was placed over the S1 ipsilateral to the lesion Intensity: 2 mA Total time: 60 minutes per session (20 minutes tDCS + 40 minutes sensorimotor training) Duration: once a day, 5 days a week, 4 weeks	Sham dual-tDCS	The IG demonstrated an increase in the FC of RPMC-SMA-LSAC relative to the CG (p<0.05)	The IG showed significantly greater improvement in FMA-UE scores compared to the CG (p<0.001)	FMA-UE changes in the IG showed a positive correlation with the FC of RPMC-SMA-LSAC (r=0.815, p<0.001)
He et al. (2025)	RCT	N=48 IG: 25 CG: 23 2 weeks to 3 months post-stroke	Modality: brain-computer interface based on motor imagery and motor attempt, combined with VR training module and robotic limb training Localization: An 8-electrode EEG system was used to acquire scalp EEG signals. The motor intention score calculated from the Fp1 electrode signal served as the modulation target for the BCI Total time: 20 minutes Duration: once a day, 5 days a week, 2 weeks Regular rehabilitation as CG	Sham BCI Regular rehabilitation, 5 days a week, 2 weeks	Following the intervention, the FC and neural activation between the SMA and other key motor-related brain regions such as PFC and M1 were enhanced (p<0.05) Post-treatment, the IG demonstrated more significant functional network reorganization compared to the CG, particularly in regions involving the LSMA with the LM1 and RPFC (p<0.05)	Compared to the CG, the IG demonstrated a significantly greater improvement in the FMA-UE scores (p=0.046)	NR
Zhang et al. (2025)	RCT	N=52 IG: 22 CG: 21 1–6 months post-stroke	Modality: anodal tDCS targeting the affected hemisphere’s leg motor area Localization: based on the international 10–20 EEG system. The anode was placed over the leg motor cortex of the affected hemisphere, slightly lateral to the Cz position. The cathode was placed over the contralateral supraorbital ridge Intensity: 2.0 mA Total time: 20 minutes Duration: 5 days per week, 4 weeks Robot-assisted gait training as CG	Robot-assisted gait training, 20 minutes, 5 days per week, 4 weeks	The IG demonstrated stronger FC between LM1 and RPMC/SMA than the CG (p=0.031)	The IG showed significantly greater improvement in FMA-LE scores compared to the CG (p<0.001)	Improved FMA-LE scores were associated with stronger FC between LM1 and RPMC/SMA, but specific correlational data were not provided
Wang et al. (2025)	Case-control study	N=28 IG: 14 stroke patients CG: 14 healthy subjects 2 weeks to 6 months post-stroke	VR upper limb rehabilitation robot training. The task was a “swatting flies” game, requiring patients to actively control the robotic arm with their paretic upper limb Duration: 10 minutes	VR upper limb rehabilitation robot training as IG	No significant difference in FC was found between the IG and CG at rest (p>0.05) Compared to rest, the CG showed no significant increase in overall FC during the task (p>0.05), but a significant increase in FC between LSMA and RPFC was observed (p<0.05) Compared to rest, the IG showed a decrease in overall FC during the task (p<0.05), with significant decreases specifically in FC between LSMA and RS1 and between LSMA and RSMA (p<0.05) During the task, the overall FC of the IG was lower than that of the CG (p<0.05), and significant differences (p<0.05) were found in FC between the following regions: LSMA-RSMA, LSMA-LM1, LSMA-RM1, LSMA-RS1, and LM1-RS1	NA	The studies proposed that VR training improves upper limb function by inducing sensorimotor network plasticity, mediated through alterations in SMA-related FC, although specific correlations with clinical scores were not reported

SMA, supplementary motor area; RCT, randomized controlled trial; IG, intervention group; CG, control group; M1, primary motor cortex; L, left; R, right; PMC, premotor cortex; S1, primary somatosensory cortex; SAC, somatosensory association cortex; PFC, prefrontal cortex; MRI, magnetic resonance imaging; rsFC, resting-state functional connectivity; GMV, grey matter volume; rTMS, repetitive transcranial magnetic stimulation; EEG, electroencephalography; RMT, resting motor threshold; tDCS, transcranial direct current stimulation; BCI, brain-computer interface; VR, virtual reality; FMA, Fugl-Meyer assessment; UE, upper extremity; LE, lower extremity; NR, not reported; NA, not applicable.

In summary, neuroimaging and neurophysiological evidence suggest that non-invasive neuromodulation techniques, or these techniques combined with motor rehabilitation, can promote motor function recovery in patients with subacute stroke by enhancing the activation and connectivity of SMA and motor-related networks. The current body of evidence is strongest for conventional techniques like rTMS and tDCS, providing a solid scientific basis for their application. Extending this evidence base to encompass and rigorously validate the effects of newer techniques such as tACS and tFUS represents a crucial future direction for the field. These methods not only reveal the dynamic process of brain network remodeling but also provide a solid scientific basis for the precise assessment and mechanistic elucidation of rehabilitation efficacy across an expanding toolkit of neuromodulation strategies.

### 2.3 The role of motor rehabilitation in the functional reorganization of the SMA

#### 2.3.1 Activation of the SMA by conventional motor rehabilitation

Conventional motor rehabilitation is widely employed in subacute stroke patients, with its core principles emphasizing repetitive and task-oriented practice, which not only helps restore impaired motor function but also significantly activates the SMA and its related neural networks (Dimyan and Cohen, 2011; Hatem et al., 2016; Peng et al., 2021). Key parameters in conventional training often involve moderate to high intensity (e.g., 60-80% of maximum voluntary contraction or perceived exertion), a frequency of 3–5 sessions per week, and a duration of 30–60 minutes per session, sustained over 4–8 weeks to induce robust neuroplastic changes (Dimyan and Cohen, 2011; Hatem et al., 2016; Peng et al., 2021). Studies show that both active and passive training methods can activate the SMA during upper limb rehabilitation, with higher activation levels observed in active training, especially at faster training speeds, where the enhancement of SMA activity is more significant (Zheng et al., 2021). This phenomenon indicates that active participation and high-intensity task-oriented training promote functional reorganization of the SMA, thereby establishing a neural foundation for enhanced rehabilitation efficacy (Zheng et al., 2021). The intensity is often graded relative to the patient’s maximum capacity, such as using 60-80% of maximum voluntary contraction (MVC) for strength tasks or challenging yet achievable speed/accuracy goals for dexterity training (Chow and Stokic, 2011; Zheng et al., 2021). Furthermore, neuroimaging studies have shown that repetitive training with 50% of the maximum voluntary contraction of the healthy hand can simultaneously activate the SMA in the damaged hemisphere and enhance its FC with motor-related brain networks (Ma et al., 2025). In subacute stroke patients, the oxygenation levels of the SMA on both the affected and contralateral sides significantly increased during this intervention, and the connectivity between the SMA and other motor areas was enhanced, suggesting the crucial pivotal role of the SMA in motor rehabilitation (Fujimoto et al., 2014; Ma et al., 2025). This demonstrates that repetitive training targeting the unaffected limb can also promote synergistic activation of both SMAs and may support the recovery of motor function on the affected side through transhemispheric neural network remodeling. In motor rehabilitation practice, emerging rehabilitation methods such as motor imagery (MI) and mirror therapy have been shown to effectively activate the SMA (Wong et al., 2013; Al-Wasity et al., 2021). MI protocols often involve 20–30 minutes of guided, kinesthetic imagery of specific movements, performed daily or several times a week (Oostra et al., 2015; Wang et al., 2026). fMRI neurofeedback studies have shown that healthy subjects can autonomously enhance the activation level of the SMA through MI combined with real-time feedback, accompanied by improved behavioral performance (Al-Wasity et al., 2021). This finding suggests that MI combined with neurofeedback not only helps patients actively regulate SMA activity but may also promote motor skill learning and functional recovery by strengthening the functional connections between the SMA and key brain regions, such as M1 (Al-Wasity et al., 2021). Furthermore, the regulatory effect of motor rehabilitation on SMA may be significantly influenced by task complexity and cognitive-motor dual-task mode. Studies have found that moderate-difficulty cognitive-motor parallel training with robot assistance can effectively induce SMA activation and enhance its network synergy (Wang et al., 2025). Specifically, robot-assisted gait training protocols often involve sessions of 30–60 minutes, administered 3–5 times per week over 4–8 weeks, with the robotic device providing adjustable levels of assistance to match patient capability (Singh et al., 2021; Wang et al., 2025). Similarly, in the subacute stroke population, a combined training regimen involving cognitive dual-task gait training and motor dual-task gait training (administered three times per week for 4 weeks) is superior to simple walking training in improving performance on obstacles, and is accompanied by enhanced SMA activity (Huimeng et al., 2025). receiving cognitive dual-task gait training and motor dual-task gait training, respectively, three times a week for 4 weeks. These findings suggest that motor rehabilitation programs that incorporate cognitive components may optimize functional reorganization by enhancing the plasticity of the SMA and its network (Huimeng et al., 2025).

In conclusion, traditional motor rehabilitation, especially those emphasizing repetitive, task-oriented, and active participation, can effectively activate the SMA and its related networks, promoting neural functional reorganization. The optimization of training parameters (e.g., intensity, frequency, duration) is fundamental to maximizing this neuroplastic response. This process provides a solid neural foundation for the recovery of motor function in subacute stroke patients and points the way for the optimization of future rehabilitation strategies.

#### 2.3.2 Motor rehabilitation promotes the reconstruction of the SMA-motor cortex network

Motor rehabilitation has shown an important role in promoting the restoration of FC between the SMA and key motor-related brain regions such as M1 and S1 in stroke patients (Rehme et al., 2011). The reconstruction of this network is highly dependent on the dose of rehabilitation, commonly defined by the total number of movement repetitions, training hours, or session frequency. Studies have used multimodal neuroimaging techniques to reveal enhanced FC between SMA, M1, and S1 after motor rehabilitation intervention (Wu et al., 2021; Zhang et al., 2025). For example, a systematic review showed that hyperbaric oxygen therapy combined with motor rehabilitation can significantly enhance the activation levels of the SMA and premotor cortex in patients with subacute stroke, and improve the FC between the SMA and areas such as S1 and the posterior parietal lobe, suggesting that this therapy helps promote functional remodeling across brain networks (Carson et al., 2005). Similarly, combining motor rehabilitation with traditional methods like acupuncture has been shown to reorganize motor-related brain regions, including SMA, M1, PMA, and the sensorimotor network, laying a neural foundation for functional recovery (Zhang et al., 2021). It can be seen that motor rehabilitation may promote the integration and functional reconstruction of the entire motor network by enhancing the synergistic activity of the SMA and the motor-sensory cortex. Further research has shown that the intensity and frequency of motor rehabilitation have a significant dose-dependent effect on SMA functional remodeling. In a study combining fNIRS and EEG, after a 4-week motor rehabilitation intervention, the strength of the FC between the SMA and M1 in patients with subacute stroke gradually increased with the continuation and intensification of rehabilitation training, and this enhancement was closely related to the improvement of motor function (Li et al., 2020). Remarkably, this study also found that the high connectivity of SMA-M1 in the early stages of motor rehabilitation may only be a functional compensation rather than a sign of true recovery (Li et al., 2020), suggesting that subsequent training must shift from pursuing connectivity strength to promoting the optimal integration of the network. Therefore, the frequency and intensity of rehabilitation training are crucial for the reconstruction of the SMA-motor cortex network, and a reasonable training program is expected to maximize the potential of neuroplasticity. In addition, different types of rehabilitation training have different effects on SMA and its related networks. For example, MI can not only improve upper limb motor function, but also promote broader neural network reorganization by enhancing the functional connections between SMA and other cognitive and sensorimotor networks (Liu et al., 2024). Studies have shown that conventional rehabilitation training mainly improves motor function through functional reorganization within the SMA, while MI tends to enhance the interaction between the SMA and other networks in the whole brain, thereby achieving a higher level of functional integration (Wang et al., 2019; Al-Wasity et al., 2021). This phenomenon suggests that composite rehabilitation training may provide a broader neural basis for the recovery of motor function. It is worth mentioning that motor-related rehabilitation training not only affects the motor cortex network of the affected limb but may also produce plasticity changes in the healthy limb and bilateral SMA and M1. Studies have shown that bilateral SMA, M1 and S1 all show decreased activation levels in the early rehabilitation stage, suggesting that the remodeling of the motor network has bilateral and staged characteristics (Culiver et al., 2025). Therefore, during rehabilitation training, full attention should be paid to the coordinated activation and network balance of bilateral brain regions to promote a more comprehensive recovery of motor function.

In summary, motor rehabilitation training can effectively promote the restoration of functional connections between the SMA and key brain regions such as M1 and S1. This reconstruction process is modulated by the dose-response effect of training intensity and frequency. Different rehabilitation modalities exhibit varying mechanisms for SMA-motor cortex network remodeling, and a combination of multiple training methods holds promise for achieving better neurological functional recovery. Future rehabilitation strategies should focus on individualized adjustments to training intensity and multi-network synergistic activation to maximize the neuroplasticity and functional reconstruction potential of subacute stroke patients.

#### 2.3.3 Clinical efficacy and neural mechanisms of motor rehabilitation

Motor rehabilitation serves as a critical component for functional recovery in subacute stroke patients, and its clinical efficacy has been extensively validated (Sale et al., 2014). Numerous randomized controlled trials and systematic reviews have demonstrated that motor rehabilitation significantly improves motor function, balance, and performance of activities of daily living in subacute stroke patients (Sale et al., 2014; Shin et al., 2022; Mehrabi et al., 2025). For instance, studies have shown that early comprehensive rehabilitation, which focuses on individualized aerobic, balance, and strength training, can significantly improve limb function, walking and balance abilities, and daily living skills in patients with subacute stroke (Jung et al., 2021). Furthermore, the introduction of emerging technologies such as virtual reality and rehabilitative robotics has diversified training modalities, thereby further improving motor outcomes and patient engagement (Kim et al., 2024). Comparative studies indicate that robot-assisted training protocols (e.g., 45-minute sessions, 3 days/week for 4–8 weeks) can lead to superior improvements in motor function and walking ability compared to conventional overground training of matched duration, with neuroimaging evidence showing enhanced activation and FC within the SMA-centered motor network (Yang et al., 2017; Li et al., 2025). Similarly, virtual reality-based rehabilitation typically employs task-specific games or simulations in sessions of 20–45 minutes, 3–5 times per week, for a similar duration, which has been shown to enhance both motor function and cognitive engagement (Torrisi et al., 2021). Long-term follow-up studies have shown that continuous motor rehabilitation has a sustained promoting effect on the functional remodeling of the SMA in subacute stroke patients, helping patients maintain and consolidate the recovery of motor function (Pirovano et al., 2023). This sustained effect is of great significance for preventing functional decline and improving the quality of life in patients with subacute stroke. It is noteworthy that rehabilitation motivation, adherence, and individualized program adaptations substantially influence treatment efficacy, underscoring the necessity of multifactorial and personalized intervention strategies (Bai and Chen, 2025).

From a neural mechanism perspective, motor rehabilitation facilitates the activation and functional reorganization of the SMA (Liu and Wang, 2022). Studies have confirmed that systematic motor rehabilitation can enhance SMA activity in patients with subacute stroke, and this enhancement is closely related to the improvement of motor function, highlighting the core mechanism of SMA in driving motor learning, exercise planning, and ultimately functional recovery (Tanaka et al., 2019; Mekbib et al., 2020; Ma et al., 2025). Neuroimaging investigations, particularly fMRI, further demonstrate that motor rehabilitation enhances FC between the lesioned and contralateral hemispheres, with strengthened interactions between the SMA, M1, and S1, and such cross-network neuroplastic changes provide a structural basis for functional restoration (Mekbib et al., 2020). For example, robot-assisted gait training has been shown to specifically enhance FC within the SMA-centered motor network, correlating with improved walking ability (Li et al., 2025). The emerging hybrid rehabilitation model further validates and refines this mechanism. A randomized controlled trial demonstrated that a brain-computer interface paradigm utilizing motor imagery and motor attempt, when integrated with virtual reality and robotic limb training, effectively enhances FC and neural activation in critical motor-related regions, including the SMA, M1, and the prefrontal cortex (He et al., 2025). These neuroplastic changes are correlated with significant functional gains in upper extremity motor performance. Another study revealed dynamic characteristics of brain network reorganization during task performance: during a virtual reality robotic task, healthy subjects exhibited a specific enhancement in connectivity between the SMA and the contralateral prefrontal cortex, whereas stroke patients demonstrated an optimization in overall FC efficiency (Wang et al., 2025). This was manifested as a reduction in widespread connections alongside a concentration of task-specific circuits, potentially reflecting an active plasticity process in which the motor network reorganizes from an inefficient, diffuse state toward a more efficient, task-specific state. These findings collectively indicate that rehabilitation based on emerging technologies can not only enhance SMA-related connectivity but also drive the network reorganization patterns towards a more efficient direction. The two representative studies outlining SMA-related neuroimaging changes and associated clinical improvements following interventions in subacute stroke are summarized in **Table 1**. Moreover, motor rehabilitation can upregulate the expression of neurotrophic factors such as brain-derived neurotrophic factor, thereby promoting synaptic plasticity and neurogenesis, which contribute to the remodeling of neural circuits (Fakhoury et al., 2022).

The neuroplastic effects of motor rehabilitation on the SMA are primarily manifested in the following aspects. Firstly, high-intensity repetitive exercise enhances neural activity and cortical excitability within the SMA, thereby improving motor planning and execution (Wang et al., 2025). Besides, motor rehabilitation facilitates the activation of residual motor pathways and their integration with sensory and cognitive networks, promoting functional compensation and redistribution of impaired abilities (Xing and Bai, 2020). Task-oriented robotic and virtual reality training, by providing intensive, repetitive practice in engaging contexts, is particularly effective at driving this integrative network plasticity (Torrisi et al., 2021; Li et al., 2025). Furthermore, motor rehabilitation induces interhemispheric functional reorganization and improves the efficiency of collateral pathway utilization, establishing a neural foundation for sustained motor recovery (Richards et al., 2008; Okabe et al., 2016). Notably, the neural mechanisms underlying motor rehabilitation extend beyond the activation of local brain regions to encompass whole-brain network reorganization, particularly through dynamic interactions between the SMA and functional networks such as the sensorimotor network and the default mode network (Chen et al., 2019; Zhang et al., 2021). Building upon these mechanisms, motor rehabilitation protocols in terms of content and intensity hold promise for further enhancing motor recovery in subacute stroke patients.

Taken together, motor rehabilitation significantly improves motor function and activities of daily living in patients with subacute stroke by promoting activation and neuroplasticity in the SMA of the key brain region. The underlying mechanisms encompass enhanced local cortical excitability, reorganization of cross-network FC, and upregulated expression of neurotrophic factors. Explicitly defined protocols are crucial for achieving consistent neuroplastic and functional outcomes. Future studies integrating multimodal assessments combining neuroimaging and biomarkers will help precisely elucidate the neural foundations of functional restoration through motor rehabilitation in subacute stroke, thereby laying the groundwork for developing more personalized and scientifically-grounded rehabilitation strategies.

### 2.4 Comparative analysis of non-invasive neuromodulation and motor rehabilitation

#### 2.4.1 Comparison of short-term and long-term efficacy between the intervention methods

Both non-invasive neuromodulation and motor rehabilitation have been demonstrated to promote functional reorganization of the SMA and related brain regions, thereby ameliorating motor dysfunction in subacute stroke sequelae (Sale et al., 2014; Stinear and Byblow, 2014; Fan et al., 2024). However, their mechanisms of action and time-dependent effects differ. Non-invasive neuromodulation focuses more on directly regulating the electrophysiological activity of neuronal networks (Romero et al., 2024; He et al., 2025; Lin et al., 2025). For instance, in animal models, it can specifically modulate impaired post-stroke gamma wave activity, improve the network dynamics of parvalbumin-positive interneurons (PV-INs), and promote the restoration of FC (Vignozzi et al., 2025). In contrast, motor rehabilitation relies more heavily on synaptic plasticity and enhanced FC induced by behavioral training (Chelette et al., 2013; Mekbib et al., 2020). Regarding short-term efficacy, non-invasive neuromodulation such as rTMS and tDCS demonstrates advantages in improving physiological indicators (Lee et al., 2026). Studies indicate that tDCS effectively enhances excitability in the M1 of subacute stroke patients, outperforming motor rehabilitation training alone in improving MEP, reflecting its potential for rapidly modulating cortical electrophysiological states and promoting neural remodeling (Kim et al., 2026; Páscoa Dos Santos et al., 2026). This direct electrophysiological modulation may underlie its significant short-term effects in improving specific motor performance. In terms of long-term functional recovery, motor rehabilitation exhibits distinct advantages. Through systematic, repetitive motor and functional task practice, it more comprehensively and stably enhances activities of daily living capacity and independence in patients with subacute stroke (Jung et al., 2021). Although TMS/tDCS demonstrates clear benefits in short-term MEP improvement, its sustained impact on long-term functional outcomes such as activities of daily living has not been fully substantiated (Lee et al., 2026). By continuously driving experience-dependent plasticity and potentially enhancing patient engagement and self-efficacy, motor rehabilitation plays an indispensable and enduring role in consolidating motor function and improving quality of life.

#### 2.4.2 Synergistic effect of the combined interventions

Notably, combined intervention with non-invasive neuromodulation and motor rehabilitation demonstrates significant synergistic effects, yielding superior outcomes across multiple dimensions compared to either intervention alone. This synergy is first mechanistically elucidated at the preclinical level. For instance, in a mouse model of stroke, combining neuromodulation of the gamma band with robotic motor training can more effectively restore the connectivity of PV-INs in the anterior motor cortex and motor-related gamma wave activity, resulting in a significant improvement in forelimb motor function (Vignozzi et al., 2025). This finding suggests that neuromodulation may prime a more plastic brain environment for subsequent experience-dependent motor training by optimizing the electrophysiological state of neural networks. Clinical research further corroborates this synergistic advantage. Multiple clinical studies indicate that combined intervention significantly enhances motor function recovery and increases activation of the SMA in patients with subacute stroke compared to single-modality therapies (Kim et al., 2010; Mitsutake et al., 2021; Soltani et al., 2026). Network meta-analyses note that while non-invasive neuromodulation techniques themselves, such as rTMS or tDCS, are effective, their combination with rehabilitation training leads to more pronounced improvements in both the speed and quality of recovery (Huang et al., 2025). More critically, the benefit of this combined strategy appears to be sustained. Clinical evidence shows that combined protocols (e.g., tDCS coupled with task-specific training) not only produce superior short-term motor gains but also maintain more persistent functional benefits and better activities of daily living at 1-month and 3-month follow-ups in subacute stroke patients (Kim et al., 2014; Hsu et al., 2023; Miranda de Aquino Miranda et al., 2025). This long-term efficacy may stem from more robust neuroplastic changes induced by the combined intervention, such as the consolidation of reorganized motor networks and their internal connectivity (e.g., within the SMA-M1 pathway). Therefore, combined therapy not only accelerates the rehabilitation process but, by leveraging the critical window of neuroplasticity in the subacute phase, also establishes a more solid foundation for patients’ functional independence. Future optimization of combined intervention strategies tailored to individual patient differences holds promise as a central direction for enhancing the efficacy of motor rehabilitation in subacute stroke.

#### 2.4.3 Analysis of mechanism differences and complementarity between the intervention methods

The mechanisms through which non-invasive neuromodulation and motor rehabilitation facilitate functional reorganization of the SMA in subacute stroke patients differ substantially. Non-invasive neuromodulation primarily achieves functional improvement by modulating cortical excitability and enhancing neural network plasticity (Cywiak et al., 2020; Huo et al., 2024). For instance, studies have shown that using tDCS at specific frequencies for post-stroke motor dysfunction can help restore local gamma wave activity, improve motor network connectivity, and enhance cortical network synchrony by modulating PV-INs circuits, thereby achieving functional reorganization (Shen et al., 2022; Vignozzi et al., 2025). Furthermore, non-invasive neuromodulation promotes functional rewiring among neurons by rectifying aberrant excitatory or inhibitory states, demonstrating particular utility during the early phases following stroke (Grefkes and Fink, 2016). In contrast, motor rehabilitation relies more heavily on behaviorally-driven neural reorganization (Richards et al., 2008; Kwakkel et al., 2023). Through repetitive active or passive movement training, it induces synaptic plasticity and functional rewiring within relevant brain regions (Dobkin, 2008; Nie et al., 2016). Animal studies have demonstrated that robot-assisted rehabilitation training can promote functional recovery in peri-lesional cortical areas and enhance motor-related neural activity (Vignozzi et al., 2025). Motor rehabilitation not only activates the motor cortex but also stimulates the reshaping of neural circuits and the formation of new synapses through continuous sensorimotor feedback (Coxon et al., 2014; Kim et al., 2018). Notably, the efficacy of motor rehabilitation often depends on training intensity, frequency, and individual active participation, with this task-oriented mechanism of neural reorganization providing a solid foundation for functional recovery in subacute patients (Huo et al., 2023). The mechanistic complementarity between these approaches provides a solid theoretical foundation for combined intervention. Studies indicate that integrating non-invasive neuromodulation with motor rehabilitation can synergistically activate multiple neuroplasticity pathways, thereby enhancing therapeutic efficacy (Kim et al., 2019; Atkinson et al., 2024). Specifically, non-invasive neuromodulation creates a plasticity window by modulating local network excitability, within which motor rehabilitation further drives the consolidation and remodeling of neural networks through repetitive behavioral tasks (Takeuchi and Izumi, 2012; Takeuchi and Izumi, 2013; Li, 2017). Experimental results show that the combined application of non-invasive neuromodulation and robot-assisted rehabilitation training can significantly improve the FC of PV-INs in the premotor cortex after stroke and effectively enhance the level of motor recovery (Vignozzi et al., 2025). This combined intervention not only restores local gamma oscillatory activity but also optimizes whole-brain-scale FC patterns, ultimately promoting maximal restoration of motor function (Vignozzi et al., 2025). Consequently, this integrated interventional strategy holds significant translational potential in clinical practice and represents a promising novel approach for advancing subacute stroke rehabilitation.

**Figure 3:**
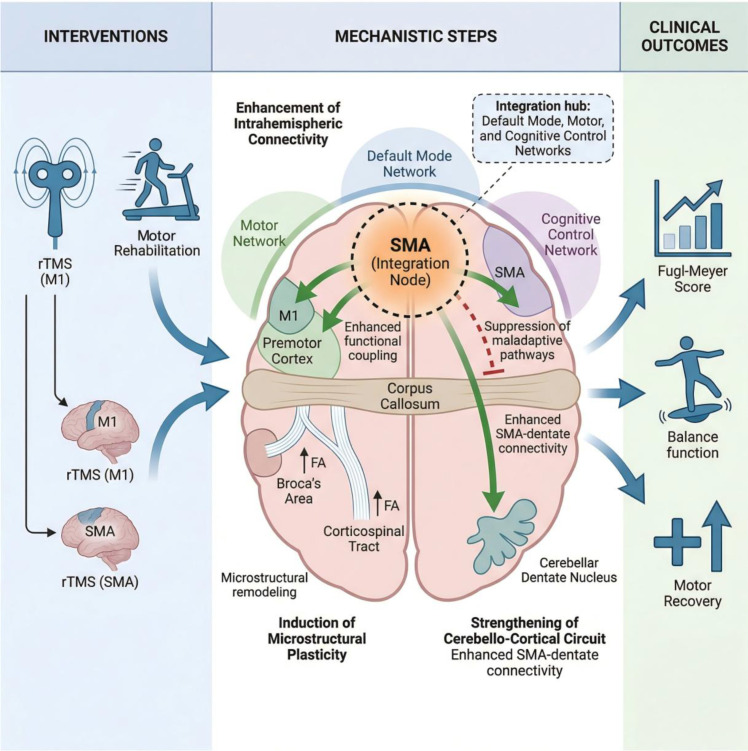
Schematic diagram of the potential mechanisms and clinical correlations of non-invasive neuromodulation and motor rehabilitation in promoting functional reorganization of the SMA in patients with subacute stroke.

In summary, non-invasive neuromodulation and motor rehabilitation each emphasize distinct mechanisms. The former directly modulates cortical excitability and network synchrony, while the latter relies on behaviorally induced remodeling of neural circuits. Their complementary characteristics provide a more comprehensive and efficient interventional strategy for the functional reorganization of the SMA after stroke. By integrating the advantages of both approaches, it is possible to further enhance rehabilitation outcomes in patients with subacute stroke, thereby offering a solid theoretical foundation for the development of personalized and precision rehabilitation protocols in the future. A schematic diagram showing the potential mechanisms and clinical correlations of non-invasive neuromodulation and motor rehabilitation in promoting SMA reorganization in subacute stroke patients is presented in **Figure 3**.

#### 2.4.4 Exploration of individualized intervention strategies

Patients with subacute stroke exhibit significant interindividual heterogeneity in the extent of injury to the SMA and their capacity for neural network reorganization (Gandolla et al., 2016), which necessitates more targeted rehabilitation intervention strategies. Using conventional, single rehabilitation programs often fails to adapt to the diversity of patients’ functional foundations, resulting in inconsistent treatment effects and significant fluctuations in recovery outcomes (Swarnakar, 2025). Therefore, individualized intervention programs for SMA injury are very important, especially for patients with subacute stroke. Individualized training should not only consider the location of the patient’s lesions, the extent of the damage, and the degree of functional impairment, but also take into account the plasticity and reconstruction potential of their neural networks (Chandrasekaran et al., 2011). For example, one study adopted an individualized protocol guided by motor control theory, by restricting the upper limb range of motion strictly below the spasticity threshold, demonstrating that this intervention significantly improved both movement quality and movement smoothness (Levin et al., 2023). In contrast, non-personalized training improves clinical scores but fails to achieve concurrent improvements in kinematic parameters. This suggests that individualized adjustments to rehabilitation programs not only aid in functional recovery but also optimize the quality of exercise execution.

Neuroimaging and neurophysiological assessments provide a scientific basis for personalized interventions. Imaging techniques allow dynamic monitoring of activation changes in the SMA and associated networks, aiding in the identification of key regions involved in functional reorganization (Lazaridou et al., 2013). For example, the fNIRS study found significant differences in the brain regions activated after rehabilitation training among different patients, with some exhibiting predominant activation in the left premotor cortex and others in S1 or right SMA (Li et al., 2022). This difference suggests that imaging assessments can help pinpoint intervention targets and optimize neuromodulation and exercise training programs. In addition, neurophysiological assessments like rTMS can be used to assess cortical excitability and network connectivity, providing a quantitative basis for individualized adjustments to intervention intensity and patterns (Rogasch and Fitzgerald, 2013). Recent research is also exploring the use of EEG and magnetic resonance electric field simulation, combined with patient anatomy and injury characteristics, to optimize personalized tDCS parameters, thereby improving the targeting and effectiveness of neuromodulation (Yoo et al., 2022).

In terms of the selection and optimization of intervention methods, various technologies such as robot-assisted rehabilitation, virtual reality, music acoustic feedback, computer games, and active and passive movement training can be incorporated into the individualized rehabilitation system. Systematic reviews and randomized controlled trials have shown that robot-assisted rehabilitation is superior to conventional rehabilitation in the recovery of motor function in patients in the subacute phase, and the efficacy is closely related to factors such as patient age, injury stage, and intervention intensity (Bhattacharjee et al., 2024; Amirbekova et al., 2025). For subacute patients with severe SMA impairment and restricted active motor function, rehabilitation predominantly relies on passive or assisted training devices, combined with neuromodulation approaches to facilitate neural network reorganization (Goto et al., 2025). In contrast, for subacute stroke patients with relatively good residual motor function, emphasis should be placed on active training, dual-task exercises, or higher-order cognitive-motor integration training to enhance the recovery of advanced motor functions (Goto et al., 2025). Consequently, the personalization of rehabilitation strategies not only involves adjustments in training content and intensity but also necessitates the judicious integration of multimodal technologies to align with the plasticity window of the patient’s neural network and functional requirements. Importantly, psychosocial factors such as patients’ subjective experiences, social support, and motor preferences can influence both the implementation and effectiveness of personalized rehabilitation interventions (Dai et al., 2024; Fu et al., 2025). Patients’ interest, engagement, and self-efficacy demonstrate strong associations with motor function recovery and long-term adherence (Dai et al., 2024). Therefore, when formulating individualized intervention plans, it is essential to comprehensively incorporate patients’ subjective preferences, living environments, and family support, while dynamically adjusting rehabilitation goals and strategies to enhance overall outcomes and quality of life.

Thus, personalized intervention strategies for functional reorganization of the SMA in patients with subacute stroke should be grounded in precise assessment, integrate neuroimaging and neurophysiological metrics, incorporate multimodal rehabilitation techniques, and address patients’ psychosocial needs. In the future, with the application of technologies such as artificial intelligence and big data, personalized and dynamically optimized rehabilitation pathways are expected to further enhance the scientific rigor and effectiveness of subacute stroke rehabilitation.

## 3 Discussion and future perspectives

The SMA constitutes a pivotal neural node for motor function recovery after stroke, with its functional reorganization playing a decisive role in determining rehabilitation outcomes. Recent investigations have progressively illuminated the central involvement of the SMA in motor recovery among patients with subacute stroke, thereby advancing both theoretical frameworks and clinical applications in neurorehabilitation. A deeper understanding of the neural mechanisms of SMA functional reorganization not only helps to clarify the pathological basis of movement disorders but also provides a scientific basis for developing precise rehabilitation intervention programs.

A comprehensive analysis of existing evidence indicates that both non-invasive neuromodulation and advanced motor rehabilitation can effectively promote functional remodeling of the SMA in patients with subacute stroke. Importantly, these approaches collectively represent a paradigm shift from traditional, supportive post-stroke interventions such as symptom-management-oriented pharmacology and conventional physical therapy focused on compensatory adaptation to mechanism-driven, network-specific interventions, demonstrating significant advantages in their mechanisms of action compared to traditional methods. Traditional drug treatments, such as antiplatelet or neurotrophic agents, primarily offer supportive recovery conditions by modulating the systemic neurochemical environment to prevent secondary damage, yet they lack spatial precision and targeted intervention in specific dysfunctional circuits like the SMA, often leading to off-target side effects (Vajda, 2002; Cook et al., 2017). In addition, while conventional physical therapy is the cornerstone of rehabilitation and focuses on improving basic motor elements such as muscle strength and joint range of motion, its mechanism of action often focuses on driving the body to produce adaptive changes or compensatory motor functions through peripheral sensory input and repetitive exercises (Albishi, 2024). However, its effectiveness in correcting specific abnormal neural networks, such as pathological interhemispheric inhibition, and achieving targeted neural circuit remodeling still has significant limitations. In contrast, the core advantage of non-invasive neuromodulation lies in its top-down, proactive, and precise ability to regulate brain circuits. For example, neuronavigated rTMS can target specific subregions of SMA, directly modulating cortical excitability to correct network imbalances (Neige et al., 2023). On the other hand, tDCS creates an initiation state for plasticity by modulating neuronal membrane potentials (Alber et al., 2017). These techniques can directly and reversibly influence the excitability and oscillatory rhythms of SMA and its connecting networks, which is difficult to achieve with drugs and conventional therapies. Emerging technologies such as tFUS promise to provide unprecedented spatial precision for non-invasive targeting of deep brain regions such as the SMA, while tACS can restore the temporal dynamics of inter-network communication by modulating specific neural oscillations such as beta waves (Zaehle et al., 2010; Jiang et al., 2025). Besides, motor rehabilitation, such as high-intensity, task-oriented robotics or virtual reality training, represents a bottom-up, experience-dependent intervention whose unique advantage lies in powerfully driving Hebbian plasticity through quantifiable, engaging exercises to specifically enhance the activation and FC of the SMA and its networks (Mazzoleni et al., 2017; Droby et al., 2020). This represents a significant advancement in the targeting and efficiency of neural remodeling compared to traditional non-targeted therapeutic exercises.

Currently, both non-invasive neuromodulation and motor rehabilitation have been shown to effectively promote functional reorganization of the SMA in patients with subacute stroke. More importantly, these two approaches are not mechanistically independent, and their strategic combination can yield synergistic effects beyond those of monotherapy, which provides a powerful multimodal strategy for subacute stroke rehabilitation to maximize functional recovery. The key to achieving this synergistic effect lies in the precise timing coordination between the two intervention modalities and the correspondence of the treatment targets, which must be guided by the specific pathophysiological state of the patient’s motor network during the subacute recovery window. Non-invasive neuromodulation optimizes motor rehabilitation by pre-regulating the brain’s network state, leveraging the principle of state-dependent plasticity to maximize plasticity induction by applying modulation during specific neural activity (Klomjai et al., 2015; Longo et al., 2022). The selection of the non-invasive neuromodulation target and protocol (excitatory *vs* inhibitory) is directly dictated by the predominant network dysfunction at a given recovery stage (Cirstea et al., 2003; Klomjai et al., 2015; Longo et al., 2022). For instance, in the early subacute phase, when pathological interhemispheric inhibition from the contralesional hemisphere often impedes recovery, applying low-frequency (inhibitory) rTMS over the contralesional M1 can dampen this maladaptive inhibition, thereby releasing the ipsilesional network and creating a permissive state for rehabilitation (Sebastianelli et al., 2017). Conversely, later in recovery or in cases of pronounced hypoexcitability of the ipsilesional SMA, applying high-frequency (excitatory) rTMS or anodal tDCS directly over the ipsilesional SMA can upregulate its excitability and enhance its FC with other motor areas, effectively priming this key hub for subsequent motor learning (Guo et al., 2021; Soltani et al., 2026). Building on this targeted approach, non-invasive neuromodulation creates such a more favorable neural environment for learning and adaptation in subsequent training by precisely regulating cortical excitability and network connectivity (Klomjai et al., 2015; Grefkes and Fink, 2016). For example, studies have confirmed that applying anodic tDCS to the SMA during balance tasks immediately improves task performance (Soltani et al., 2026). Similarly, combining anodic tDCS with weight-bearing gait training is more effective in improving gait in subacute stroke patients than gait training alone (Mitsutake et al., 2021). The underlying neural mechanism may involve the immediate enhancement of SMA-related network connectivity by tDCS, which provides an optimized network basis for the encoding and consolidation of motor skills (Mitsutake et al., 2021; Soltani et al., 2026). This top-down network modulation, combined with the bottom-up experience-driven approach of motor rehabilitation, may accelerate the motor learning process (Emara et al., 2010). While motor rehabilitation itself can enhance and prolong the neuroplasticity induced by non-invasive neuromodulation, thereby improving its persistence (Mazzoleni et al., 2017; Droby et al., 2020). Changes in cortical excitability induced by non-invasive neuromodulation like tDCS are usually temporary (Impey et al., 2017). However, when non-invasive modulation is combined with behaviorally relevant, repetitive motor training, the synaptic changes induced in the initial stages of modulation can be selectively reinforced through task-induced specific neural activity, based on the Hebbian learning mechanism, thereby guiding them into stable, adaptive functional circuit reorganization (Narayana et al., 2014; Bjørndal et al., 2024). Without this task-related neural exercise, the changes induced by non-invasive neuromodulation may tend to be nonspecific or transient (Bjørndal et al., 2024). Conversely, motor rehabilitation training performed in a regulated or optimized brain network state may more efficiently drive synaptic strengthening and network remodeling in SMA and related motor cortex (Malerba et al., 2017). The timing of motor rehabilitation relative to non-invasive neuromodulation is thus crucial, as training conducted during or shortly after the excitability modulation capitalizes on the primed state to drive specific, adaptive plasticity (Narayana et al., 2014; Bjørndal et al., 2024). Studies have shown that combining anodic tDCS targeting SMA with task-oriented training such as weight-loss walking training not only produces better motor function outcomes compared to any single intervention but may also be accompanied by enhanced and sustained FC within the SMA-M1 network (Mitsutake et al., 2021; Zhang et al., 2025; Soltani et al., 2026). Furthermore, the sensory feedback and successful experiences provided by motor rehabilitation training may further promote structural remodeling of neural circuits related to the regulatory target, thereby transforming transient excitatory modulation into lasting changes in neural connectivity (Bolognini et al., 2016). Therefore, for subacute stroke patients, designing individualized and time-optimized combined intervention strategies through multimodal assessment is key to maximizing functional recovery potential. This entails selecting the neuromodulation target (e.g., contralesional M1 *vs* ipsilesional SMA/pre-SMA) and mode based on individualized neuroimaging/neurophysiological assessment of network pathology, and precisely timing the delivery of targeted motor rehabilitation to coincide with the window of optimized cortical excitability. Future research should systematically optimize the parameters of combined intervention programs, including this critical target-time correspondence, and explore advanced strategies such as multi-target network modulation to fully unleash their therapeutic potential.

In evaluating and comparing the efficacy of different interventions (single or combined) on SMA functional reorganization, it is crucial to consider a spectrum of potential confounding factors that may significantly influence therapeutic success. These factors introduce heterogeneity in treatment response and must be accounted for when interpreting outcomes. Firstly, baseline neuropathological and patient-specific characteristics are fundamental determinants. The precise location and extent of the stroke lesion directly affect the structural integrity of the SMA and its connectivity with key nodes like the primary motor and premotor cortices, thereby modulating its responsiveness to intervention (Lazaridou et al., 2013). The degree of pathological interhemispheric inhibition, a critical network state, dictates the rationale for specific neuromodulation approaches (Fan et al., 2026). Furthermore, factors such as age, pre-stroke brain reserve, baseline stroke severity, time since stroke onset within the subacute window, and the presence of comorbidities (e.g., diabetes, hypertension, cognitive impairment) can all modulate the capacity for neural reorganization and functional recovery. Besides, intervention-specific parameters are pivotal sources of variability. For non-invasive neuromodulation, efficacy is highly dependent on stimulation intensity, frequency, pattern, duration, and, crucially, targeting precision (e.g., targeting SMA proper versus pre-SMA) (Hamada et al., 2008; Shirota et al., 2013; Flamez et al., 2021; Kahl et al., 2021). Inaccurate neuronavigation or anatomical variability can lead to suboptimal electric field distribution over the SMA (Soltani et al., 2026). For motor rehabilitation, the type (e.g., robotic, task-specific), intensity, frequency, and overall dose of training, as well as patient adherence and motivation, are well-established modulators of experience-dependent plasticity (Dimyan and Cohen, 2011; Hatem et al., 2016; Peng et al., 2021). In the context of combined interventions, the timing and sequence of application (e.g., neuromodulation as a primer before training versus concurrent application) are additional critical variables that may determine the magnitude of synergistic effects (Kim et al., 2010; Mitsutake et al., 2021; Soltani et al., 2026). Additionally, methodological differences, including sample size, control group design (e.g., sham stimulation), blinding, selection of primary outcome measures (e.g., combining clinical scales with neurophysiological indicators), and follow-up time, directly affect the interpretation of efficacy comparison results and can partially explain the differences in reported results between single-method and combined-method approaches (Hampsey et al., 2026). Critically, the influence of these confounders may differ between single and combined interventions. Combined protocols, by integrating two distinct mechanisms of action, may have a narrower therapeutic window for optimal parameterization and might be more sensitive to suboptimal dosing or mistiming. Conversely, they may also offer greater robustness against certain patient-specific factors (e.g., low intrinsic plasticity) by providing a stronger drive for reorganization. Future research and clinical translation must therefore move beyond one-size-fits-all protocols. Leveraging multimodal neurophysiological and imaging assessments to characterize these confounding factors will be essential for developing stratified, personalized, and dynamically adjusted intervention strategies to maximize the precision and efficacy of SMA-targeted therapies in subacute stroke.

Looking ahead, integrating the latest technologies will usher in a new era of precision and closed-loop neurorehabilitation. The limitations of current evidence, such as incompletely elucidated mechanisms, lack of standardized parameters, and unclear impact of individual differences, are key areas for future research. Firstly, biomarker-guided individualized strategies should be developed. For example, developing closed-loop neuromodulation systems to dynamically adjust tACS or tDCS parameters based on SMA involvement feedback from real-time EEG, and utilizing tFUS-mediated targeted delivery technology to precisely deliver neurotrophic factors to the SMA surrounding the lesion to enhance local plasticity. Furthermore, multimodal neuroimaging such as fMRI and DTI, along with machine learning methods, are needed to deeply analyze the dynamic process of SMA functional reorganization and establish predictive models of patient baseline characteristics such as lesion topology, genetic markers, connectomics, and intervention responses to build a robust framework for personalized intervention. Additionally, clinical practice needs to strengthen interdisciplinary collaboration, integrating the strengths of neuroscience, rehabilitation medicine, and engineering technologies to optimize rehabilitation pathways and improve treatment outcomes and quality of life for patients with subacute stroke.

In conclusion, SMA functional reconstruction is a core component of motor function recovery in subacute stroke. While traditional methods form the basis of care, non-invasive neuromodulation and advanced motor rehabilitation offer transformative therapeutic potential through their core mechanisms of direct, circuit-specific, and high-dose guided plasticity. By continuously deepening research into the mechanisms of SMA functional remodeling and committing to developing synergistic, technology-enhanced, and individualized rehabilitation strategies, future neurorehabilitation interventions are expected to become more precise and effective, ultimately significantly improving motor function and quality of life in subacute stroke patients.

## References

[B1] Abidi M. de Marco G. Couillandre A. Feron M. Mseddi E. Termoz N. . (2020). Adaptive functional reorganization in amyotrophic lateral sclerosis: coexisting degenerative and compensatory changes. Eur. J. Neurol. 27, 121–128. doi: 10.1111/ene.14042. PMID: 31310452

[B2] Afridi A. Malik A. N. Rathore F. A. (2023). Task oriented training for stroke rehabilitation: a mini review. J. Pak. Med. Assoc. 73, 2295–2297. doi: 10.47391/jpma.23-98. PMID: 38013554

[B3] Alario F. X. Chainay H. Lehericy S. Cohen L. (2006). The role of the supplementary motor area (SMA) in word production. Brain Res. 1076, 129–143. doi: 10.1016/j.brainres.2005.11.104. PMID: 16480694

[B4] Alber R. Moser H. Gall C. Sabel B. A. (2017). Combined transcranial direct current stimulation and vision restoration training in subacute stroke rehabilitation: A pilot study. Pm R. 9, 787–794. doi: 10.1016/j.pmrj.2016.12.003. PMID: 28082176

[B5] Albishi A. M. (2024). Knowledge, attitudes, and perceptions of physical therapists towards conventional physical therapy-across-sectional study. Ann. Med. Surg. (Lond) 86, 1942–1949. doi: 10.1097/ms9.0000000000001883. PMID: 38576907 PMC10990403

[B6] Al-Wasity S. Vogt S. Vuckovic A. Pollick F. E. (2021). Upregulation of supplementary motor area activation with fMRI neurofeedback during motor imagery. eNeuro 8, ENEURO.0377-18.2020. doi: 10.1523/eneuro.0377-18.2020. PMID: 33376115 PMC7877466

[B7] Amirbekova M. Kispayeva T. Adomaviciene A. Eszhanova L. Bolshakova I. Ospanova Z. (2025). Systematic review and meta-analysis of effectiveness of robotic therapy in the recovery of motor functions after stroke. Front. Hum. Neurosci. 19. doi: 10.3389/fnhum.2025.1622661. PMID: 40761519 PMC12318999

[B8] Atkinson D. Barta K. Bizama F. Anderson H. Brose S. Sayenko D. G. (2024). Transcutaneous spinal stimulation combined with locomotor training improves functional outcomes in a child with cerebral palsy: A case study. Children (Basel) 11, 1439. doi: 10.3390/children11121439. PMID: 39767868 PMC11675040

[B9] Avan A. Digaleh H. Di Napoli M. Stranges S. Behrouz R. Shojaeianbabaei G. . (2019). Socioeconomic status and stroke incidence, prevalence, mortality, and worldwide burden: an ecological analysis from the Global Burden of Disease Study 2017. BMC Med. 17, 1–30. doi: 10.1186/s12916-019-1397-3. PMID: 31647003 PMC6813111

[B10] Badran B. W. Peng X. Baker-Vogel B. Hutchison S. Finetto P. Rishe K. . (2023). Motor activated auricular vagus nerve stimulation as a potential neuromodulation approach for post-stroke motor rehabilitation: a pilot study. Neurorehabil. Neural Repair 37, 374–383. doi: 10.1177/15459683231173357. PMID: 37209010 PMC10363288

[B11] Bai J. Chen K. (2025). Advances in nursing care for post-stroke limb dysfunction rehabilitation. Front. Neurol. 16. doi: 10.3389/fneur.2025.1615500. PMID: 40777855 PMC12329226

[B12] Bhat P. Kumaran S. S. Goyal V. Srivastava A. K. Behari M. (2023). Effect of rTMS at SMA on task-based connectivity in PD. Behav. Brain Res. 452, 114602. doi: 10.1016/j.bbr.2023.114602. PMID: 37516209

[B13] Bhattacharjee S. Barman A. Patel S. Sahoo J. (2024). The combined effect of robot-assisted therapy and activities of daily living training on upper limb recovery in persons with subacute stroke: A randomized controlled trial. Arch. Phys. Med. Rehabil. 105, 1041–1049. doi: 10.1016/j.apmr.2024.01.027. PMID: 38367830

[B14] Bjørndal J. R. Beck M. M. Jespersen L. Christiansen L. Lundbye-Jensen J. (2024). Hebbian priming of human motor learning. Nat. Commun. 15, 5126. doi: 10.1038/s41467-024-49478-5. PMID: 38879614 PMC11180091

[B15] Bolognini N. Russo C. Edwards D. J. (2016). The sensory side of post-stroke motor rehabilitation. Restor. Neurol. Neurosci. 34, 571–586. doi: 10.3233/rnn-150606. PMID: 27080070 PMC5605470

[B16] Bonanno M. De Luca R. De Nunzio A. M. Quartarone A. Calabrò R. S. (2022). Innovative technologies in the neurorehabilitation of traumatic brain injury: a systematic review. Brain Sci. 12, 1678. doi: 10.3390/brainsci12121678. PMID: 36552138 PMC9775990

[B17] Braaß H. Gutgesell L. Backhaus W. Higgen F. L. Quandt F. Choe C. U. . (2023). Early functional connectivity alterations in contralesional motor networks influence outcome after severe stroke: a preliminary analysis. Sci. Rep. 13, 11010. doi: 10.1038/s41598-023-38066-0. PMID: 37419966 PMC10328915

[B18] Bruyn N. Bonkhoff A. K. Saenen L. Thijs L. Essers B. Alaerts K. . (2023). Altered dynamic resting state functional connectivity associated with somatosensory impairments in the upper limb in the early sub-acute phase post-stroke. Neurorehabil. Neural Repair 37, 423–433. doi: 10.1177/15459683231179172. PMID: 37350441

[B19] Butchbach M. E. R. Scott R. C. (2022). Biological networks and complexity in early-onset motor neuron diseases. Front. Neurol. 13. doi: 10.3389/fneur.2022.1035406. PMID: 36341099 PMC9634177

[B20] Cao P. Guo S. Zhang G. Zan X. Wang J. Zhang F. . (2025). Brain-computer interface training for multimodal functional recovery in patients with brain injury: A case series. Quant. Imaging Med. Surg. 15, 9277–9293. doi: 10.21037/qims-2025-1136. PMID: 41081225 PMC12514666

[B21] Carlsen A. N. Eagles J. S. MacKinnon C. D. (2015). Transcranial direct current stimulation over the supplementary motor area modulates the preparatory activation level in the human motor system. Behav. Brain Res. 279, 68–75. doi: 10.1016/j.bbr.2014.11.009. PMID: 25446764 PMC4857713

[B22] Carson S. McDonagh M. Russman B. Helfand M. (2005). Hyperbaric oxygen therapy for stroke: a systematic review of the evidence. Clin. Rehabil. 19, 819–833. doi: 10.1191/0269215505cr907oa. PMID: 16323381

[B23] Catalogna M. Hadanny A. Parag Y. Adler M. Elkarif V. Efrati S. (2023). Functional MRI evaluation of hyperbaric oxygen therapy effect on hand motor recovery in a chronic post-stroke patient: a case report and physiological discussion. Front. Neurol. 14. doi: 10.3389/fneur.2023.1233841. PMID: 37840920 PMC10570419

[B24] Catani M. Dell'acqua F. Vergani F. Malik F. Hodge H. Roy P. . (2012). Short frontal lobe connections of the human brain. Cortex 48, 273–291. doi: 10.1016/j.cortex.2011.12.001. PMID: 22209688

[B25] Censor N. Dayan E. Cohen L. G. (2014). Cortico-subcortical neuronal circuitry associated with reconsolidation of human procedural memories. Cortex 58, 281–288. doi: 10.1016/j.cortex.2013.05.013. PMID: 23849672 PMC3873366

[B26] Ceradini M. Losanno E. Micera S. Bandini A. Orlandi S. (2024). Immersive VR for upper-extremity rehabilitation in patients with neurological disorders: a scoping review. J. NeuroEng. Rehabil. 21, 75. doi: 10.1186/s12984-024-01367-0. PMID: 38734690 PMC11088157

[B27] Chandrasekaran S. Ament S. A. Eddy J. A. Rodriguez-Zas S. L. Schatz B. R. Price N. D. . (2011). Behavior-specific changes in transcriptional modules lead to distinct and predictable neurogenomic states. Proc. Natl. Acad. Sci. U.S.A. 108, 18020–18025. doi: 10.1073/pnas.1114093108. PMID: 21960440 PMC3207651

[B28] Chelette K. C. Carrico C. Nichols L. Sawaki L. (2013). Long-term cortical reorganization following stroke in a single subject with severe motor impairment. NeuroRehabilitation 33, 385–389. doi: 10.3233/nre-130968. PMID: 23949080

[B29] Chen J. Fan Y. Jia X. Fan F. Wang J. Zou Q. . (2025). The supplementary motor area as a flexible hub mediating behavioral and neuroplastic changes in motor sequence learning: A TMS and TMS-EEG study. Neurosci. Bull. 41, 837–852. doi: 10.1007/s12264-025-01375-7. PMID: 40080252 PMC12014987

[B30] Chen C. Fang Y. Wang X. Bao S. C. Tang Z. Tong R. K. (2019). Excitation comparison between multi-site stimulation using network-based tDCS and focal stimulation using high-definition tDCS. Annu. Int. Conf. IEEE Eng. Med. Biol. Soc 2019, 6884–6887. doi: 10.1109/embc.2019.8857287. PMID: 31947422

[B31] Chen J. Liu M. Sun D. Jin Y. Wang T. Ren C. (2018). Effectiveness and neural mechanisms of home-based telerehabilitation in patients with stroke based on fMRI and DTI: a study protocol for a randomized controlled trial. Med. (Baltimore) 97, e9605. doi: 10.1097/md.0000000000009605. PMID: 29504985 PMC5779754

[B32] Chen N. Qiu X. Hua Y. Hu J. Bai Y. (2023). Effects of sequential inhibitory and facilitatory repetitive transcranial magnetic stimulation on neurological and functional recovery of a patient with chronic stroke: a case report and literature review. Front. Neurol. 14. doi: 10.3389/fneur.2023.1064718. PMID: 36779047 PMC9911674

[B33] Chen H. Shi M. Zhang H. Zhang Y. D. Geng W. Jiang L. . (2019). Different patterns of functional connectivity alterations within the default-mode network and sensorimotor network in basal ganglia and pontine stroke. Med. Sci. Monit. 25, 9585–9593. doi: 10.12659/msm.918185. PMID: 31838483 PMC6929567

[B34] Chieffo R. Comi G. Leocani L. (2016). Noninvasive neuromodulation in poststroke gait disorders: rationale, feasibility, and state of the art. Neurorehabil. Neural Repair 30, 71–82. doi: 10.1177/1545968315586464. PMID: 25967759

[B35] Chow A. D. Shin J. Wang H. Kellawan J. M. Pereira H. M. (2022). Influence of transcranial direct current stimulation dosage and associated therapy on motor recovery post-stroke: A systematic review and meta-analysis. Front. Aging Neurosci. 14. doi: 10.3389/fnagi.2022.821915. PMID: 35370603 PMC8972130

[B36] Chow J. W. Stokic D. S. (2011). Force control of quadriceps muscle is bilaterally impaired in subacute stroke. J. Appl. Physiol. (1985) 111, 1290–1295. doi: 10.1152/japplphysiol.00462.2011. PMID: 21885803

[B37] Chu C. S. Lin Y. Y. Huang C. C. Chung Y. A. Park S. Y. Chang W. C. . (2025). Comparing different montages of transcranial direct current stimulation in treating treatment-resistant obsessive compulsive disorder: A randomized, single-blind clinical trial. Med. (Kaunas) 61, 169. doi: 10.3390/medicina61020169. PMID: 40005287 PMC11857099

[B38] Cirstea M. C. Mitnitski A. B. Feldman A. G. Levin M. F. (2003). Interjoint coordination dynamics during reaching in stroke. Exp. Brain Res. 151, 289–300. doi: 10.1007/s00221-003-1438-0. PMID: 12819841

[B39] Coelho D. B. Aquino Dos Santos A. C. Sato J. R. Simis M. Fregni F. Battistella L. R. (2025). Neurorehabilitation in spinal cord injury: Increased cortical activity through tDCS and robotic gait training. Clin. Neurophysiol. 173, 199–204. doi: 10.1016/j.clinph.2025.03.027. PMID: 40153922

[B40] Cook D. J. Nguyen C. Chun H. N. I L. L. Chiu A. S. Machnicki M. . (2017). Hydrogel-delivered brain-derived neurotrophic factor promotes tissue repair and recovery after stroke. J. Cereb. Blood Flow Metab. 37, 1030–1045. doi: 10.1177/0271678x16649964. PMID: 27174996 PMC5363479

[B41] Coxon J. P. Peat N. M. Byblow W. D. (2014). Primary motor cortex disinhibition during motor skill learning. J. Neurophysiol. 112, 156–164. doi: 10.1152/jn.00893.2013. PMID: 24717346

[B42] Culiver A. M. Grooms D. R. Caccese J. B. Hayes S. M. Schmitt L. C. Oñate J. A. (2025). fMRI activation in sensorimotor regions at 6 weeks after anterior cruciate ligament reconstruction. Am. J. Sports Med. 53, 791–800. doi: 10.1177/03635465251313808. PMID: 39905651 PMC12273877

[B43] Cywiak C. Ashbaugh R. C. Metto A. C. Udpa L. Qian C. Gilad A. A. . (2020). Non-invasive neuromodulation using rTMS and the electromagnetic-perceptive gene (EPG) facilitates plasticity after nerve injury. Brain Stimul 13, 1774–1783. doi: 10.1016/j.brs.2020.10.006. PMID: 33068795 PMC7722061

[B44] Dai Y. Shi H. Ji K. Han Y. De Ala M. Wang Q. (2024). Exercise preference in stroke survivors: a concept analysis. Front. Neurol. 15. doi: 10.3389/fneur.2024.1326649. PMID: 38414548 PMC10896848

[B45] Dali G. Brosnan M. Tiego J. Johnson B. P. Fornito A. Bellgrove M. A. . (2022). Examining the neural correlates of error awareness in a large fMRI study. Cereb. Cortex 33, 458–468. doi: 10.1093/cercor/bhac077. PMID: 35238340 PMC9837605

[B46] Davidson B. Bhattacharya A. Sarica C. Darmani G. Raies N. Chen R. . (2024). Neuromodulation techniques - from non-invasive brain stimulation to deep brain stimulation. Neurotherapeutics 21, e00330. doi: 10.1016/j.neurot.2024.e00330. PMID: 38340524 PMC11103220

[B47] Dimyan M. A. Cohen L. G. (2011). Neuroplasticity in the context of motor rehabilitation after stroke. Nat. Rev. Neurol. 7, 76–85. doi: 10.1038/nrneurol.2010.200. PMID: 21243015 PMC4886719

[B48] Ding X. Zhang S. Huang W. Zhang S. Zhang L. Hu J. . (2022). Comparative efficacy of non-invasive brain stimulation for post-stroke aphasia: a network meta-analysis and meta-regression of moderators. Neurosci. Biobehav. Rev. 140, 104804. doi: 10.1016/j.neubiorev.2022.104804. PMID: 35926728

[B49] Dobkin B. H. (2008). Fatigue versus activity-dependent fatigability in patients with central or peripheral motor impairments. Neurorehabil. Neural Repair 22, 105–110. doi: 10.1177/1545968308315046. PMID: 18285599 PMC4160309

[B50] Droby A. Maidan I. Jacob Y. Giladi N. Hausdorff J. M. Mirelman A. (2020). Distinct effects of motor training on resting-state functional networks of the brain in Parkinson's disease. Neurorehabil. Neural Repair 34, 795–803. doi: 10.1177/1545968320940985. PMID: 32684069

[B51] Du X. Guo X. Zhou X. (2025). Recovery of nonketotic hyperglycaemic hemichorea -hemiballismus due to acute ischemic stroke in the contralateral supplementary motor area: a case report and literature review. Folia Neuropathol. 63, 100–105. doi: 10.5114/fn.2024.135290. PMID: 39165208

[B52] Du J. Yang F. Zhang Z. Hu J. Xu Q. Hu J. . (2018). Early functional MRI activation predicts motor outcome after ischemic stroke: a longitudinal, multimodal study. Brain Imaging Behav. 12, 1804–1813. doi: 10.1007/s11682-018-9851-y. PMID: 29766355

[B53] Dyke K. Jackson G. M. Nixon E. Jackson S. R. (2019). Effects of single-session cathodal transcranial direct current stimulation on tic symptoms in Tourette's syndrome. Exp. Brain Res. 237, 2853–2863. doi: 10.1007/s00221-019-05637-5. PMID: 31463531 PMC6794240

[B54] Emara T. H. Moustafa R. R. ElNahas N. M. ElGanzoury A. M. Abdo T. A. Mohamed S. A. . (2010). Repetitive transcranial magnetic stimulation at 1Hz and 5Hz produces sustained improvement in motor function and disability after ischaemic stroke. Eur. J. Neurol. 17, 1203–1209. doi: 10.1111/j.1468-1331.2010.03000.x. PMID: 20402755

[B55] Ertelt D. Small S. Solodkin A. Dettmers C. McNamara A. Binkofski F. . (2007). Action observation has a positive impact on rehabilitation of motor deficits after stroke. Neuroimage 36, T164–T173. doi: 10.1016/j.neuroimage.2007.03.043. PMID: 17499164

[B56] Esht V. Alshehri M. M. Balasubramanian K. Sanjeevi R. R. Shaphe M. A. Alhowimel A. . (2024). Transcranial direct current stimulation (tDCS) for neurological disability among subacute stroke survivors to improve multiple domains in health-related quality of life: randomized controlled trial protocol. Neurophysiol. Clinique 54, 102976. doi: 10.1016/j.neucli.2024.102976. PMID: 38663043

[B57] Fakhoury M. Eid F. El Ahmad P. Khoury R. Mezher A. El Masri D. . (2022). Exercise and dietary factors mediate neural plasticity through modulation of BDNF signaling. Brain Plast. 8, 121–128. doi: 10.3233/bpl-220140. PMID: 36448042 PMC9661351

[B58] Fan H. Wang H. Lian Z. Yu Q. Wu X. Kuang N. . (2026). Dynamic interactions between hemispheres reveal a compensatory pathway for motor recovery in moderate-to-severe subcortical stroke. J. Stroke 28, 97–114. doi: 10.5853/jos.2025.01725. PMID: 41478717 PMC12883883

[B59] Fan S. Yan L. Zhang J. Qian Y. Wang M. Yang L. . (2024). Effects of repetitive transcranial magnetic stimulation on lower extremity motor function and optimal parameters in stroke patients with different stages of stroke: a systematic evaluation and meta-analysis. Front. Neurol. 15. doi: 10.3389/fneur.2024.1372159. PMID: 39131051 PMC11310066

[B60] Feitosa J. A. Fernandes C. A. Casseb R. F. Castellano G. (2022). Effects of virtual reality-based motor rehabilitation: a systematic review of fMRI studies. J. Neural Eng. 19. doi: 10.1088/1741-2552/ac456e. PMID: 34933281

[B61] Flamez A. Wu G. R. Wiels W. Van Schuerbeek P. De Mey J. De Keyser J. . (2021). Opposite effects of one session of 1 Hz rTMS on functional connectivity between pre-supplementary motor area and putamen depending on the dyskinesia state in Parkinson's disease. Clin. Neurophysiol. 132, 851–856. doi: 10.1016/j.clinph.2020.12.024. PMID: 33636601

[B62] Fortier-Lebel N. Nakajima T. (2025). Exploring the consistent roles of motor areas across voluntary movement and locomotion. Neuroscientist 31, 279–295. doi: 10.1177/10738584241263758. PMID: 39041460 PMC12103638

[B63] Fu J. Zhu J. Dong J. Wang Y. Wang S. Zhang X. . (2025). Recovery from severe mental illnesses: The influence of personal and psychosocial factors in community settings. Int. J. Ment. Health Nurs. 34, e13440. doi: 10.1111/inm.13440. PMID: 39334331

[B64] Fujimoto H. Mihara M. Hattori N. Hatakenaka M. Kawano T. Yagura H. . (2014). Cortical changes underlying balance recovery in patients with hemiplegic stroke. Neuroimage 85, 547–554. doi: 10.1016/j.neuroimage.2013.05.014. PMID: 23684871

[B65] Gandolla M. Ward N. S. Molteni F. Guanziroli E. Ferrigno G. Pedrocchi A. (2016). The neural correlates of long-term carryover following functional electrical stimulation for stroke. Neural Plast. 2016, 4192718. doi: 10.1155/2016/4192718. PMID: 27073701 PMC4814690

[B66] Goto K. Kawasaki T. Hamada H. Sihan Lim V. Shimada R. Nakazato R. . (2025). Changes in gait metrics and motor strategies post-neurocognitive rehabilitation in subacute stroke: A retrospective mixed-methods study. Cureus 17, e86264. doi: 10.7759/cureus.86264. PMID: 40693099 PMC12277929

[B67] Gould L. Kress S. Neudorf J. Gibb K. Persad A. Meguro K. . (2021). An fMRI, DTI and neurophysiological examination of atypical organization of motor cortex in ipsilesional hemisphere following post-stroke recovery. J. Stroke Cerebrovasc. Dis. 30, 105593. doi: 10.1016/j.jstrokecerebrovasdis.2020.105593. PMID: 33434816

[B68] Grados M. Huselid R. Duque-Serrano L. (2018). Transcranial magnetic stimulation in Tourette syndrome: a historical perspective, its current use and the influence of comorbidities in treatment response. Brain Sci. 8, 129. doi: 10.3390/brainsci8070129. PMID: 29986411 PMC6071080

[B69] Grefkes C. Fink G. R. (2016). Noninvasive brain stimulation after stroke: it is time for large randomized controlled trials! Curr. Opin. Neurol. 29, 714–720. doi: 10.1097/wco.0000000000000395. PMID: 27648877

[B70] Grefkes C. Nowak D. A. Wang L. E. Dafotakis M. Eickhoff S. B. Fink G. R. (2010). Modulating cortical connectivity in stroke patients by rTMS assessed with fMRI and dynamic causal modeling. Neuroimage 50, 233–242. doi: 10.1016/j.neuroimage.2009.12.029. PMID: 20005962 PMC8020334

[B71] Guo Z. Gong Y. Lu H. Qiu R. Wang X. Zhu X. . (2022). Multitarget high-definition transcranial direct current stimulation improves response inhibition more than single-target high-definition transcranial direct current stimulation in healthy participants. Front. Neurosci. 16. doi: 10.3389/fnins.2022.905247. PMID: 35968393 PMC9372262

[B72] Guo Z. Jin Y. Bai X. Jiang B. He L. McClure M. A. . (2021). Distinction of high- and low-frequency repetitive transcranial magnetic stimulation on the functional reorganization of the motor network in stroke patients. Neural Plast. 2021, 8873221. doi: 10.1155/2021/8873221. PMID: 33542729 PMC7840259

[B73] Hamada M. Ugawa Y. Tsuji S. (2008). High-frequency rTMS over the supplementary motor area for treatment of Parkinson's disease. Mov. Disord. 23, 1524–1531. doi: 10.1002/mds.22168. PMID: 18548577

[B74] Hampsey E. Kalfas M. Carter L. Bloomfield M. Rezaei H. Young A. H. . (2026). A systematic review of transcranial electrical stimulation and meta-analysis of transcranial direct current stimulation RCTs in unipolar and bipolar depression. J. Affect. Disord. 400, 121009. doi: 10.1016/j.jad.2025.121009. PMID: 41520839

[B75] Han X. Bai L. Sun C. Niu X. Ning Y. Chen Z. . (2019). Acupuncture enhances communication between cortices with damaged white matters in poststroke motor impairment. Evid. Based Complement. Alternat. Med. 2019, 4245753. doi: 10.1155/2019/4245753. PMID: 30719060 PMC6334314

[B76] Han Y. Jing Y. Shi Y. Mo H. Wan Y. Zhou H. . (2024). The role of language-related functional brain regions and white matter tracts in network plasticity of post-stroke aphasia. J. Neurol. 271, 3095–3115. doi: 10.1007/s00415-024-12358-5. PMID: 38607432

[B77] Han X. Shen J. Chen S. Cai Z. Zhu Y. Yi W. . (2023). Ultrasonic-controlled “explosive” hydrogels to precisely regulate spatiotemporal osteoimmune disturbance. Biomaterials 295, 122057. doi: 10.1016/j.biomaterials.2023.122057. PMID: 36805244

[B78] Han X. Wang F. Xiao P. Yang Z. Liu M. Han Z. . (2025). Electrosensitive heterogeneous short fibers via acousto‐electric coupling for sequential bone regeneration in infectious defects. Adv. Sci. 12, e14174. doi: 10.1002/advs.202514174. PMID: 41082442 PMC12752602

[B79] Hanakawa T. Hotta F. Nakamura T. Shindo K. Ushiba N. Hirosawa M. . (2023). Macrostructural cerebellar neuroplasticity correlates with motor recovery after stroke. Neurorehabil. Neural Repair 37, 775–785. doi: 10.1177/15459683231207356. PMID: 37882368

[B80] Hannanu F. F. Zeffiro T. A. Lamalle L. Heck O. Renard F. Thuriot A. . (2017). Parietal operculum and motor cortex activities predict motor recovery in moderate to severe stroke. NeuroImage Clin. 14, 518–529. doi: 10.1016/j.nicl.2017.01.023. PMID: 28317947 PMC5342999

[B81] Hasui N. Mizuta N. Taguchi J. Nakatani T. Morioka S. (2022). Effects of transcranial direct current stimulation over the supplementary motor area combined with walking on the intramuscular coherence of the tibialis anterior in a subacute post-stroke patient: A single-case study. Brain Sci. 12, 540. doi: 10.3390/brainsci12050540. PMID: 35624929 PMC9139188

[B82] Hatem S. M. Saussez G. Della Faille M. Prist V. Zhang X. Dispa D. . (2016). Rehabilitation of motor function after stroke: A multiple systematic review focused on techniques to stimulate upper extremity recovery. Front. Hum. Neurosci. 10. doi: 10.3389/fnhum.2016.00442. PMID: 27679565 PMC5020059

[B83] He M. Wu P. Long X. Shi C. Cao X. Huang X. . (2025). Efficacy of low-intensity transcranial ultrasound stimulation in enhancing motor recovery via modulation of microglial polarization, angiogenesis and neurogenesis in MCAO rats. Ultrasound Med. Biol. 51, 1915–1924. doi: 10.1016/j.ultrasmedbio.2025.05.031. PMID: 40796499

[B84] He J. Yuan Z. Quan L. Xi H. Guo J. Zhu D. . (2025). Multimodal assessment of a BCI system for stroke rehabilitation integrating motor imagery and motor attempts: a randomized controlled trial. J. NeuroEng. Rehabil. 22, 185. doi: 10.1186/s12984-025-01723-8. PMID: 40859358 PMC12379318

[B85] Hirabayashi R. Kojima S. Edama M. Onishi H. (2020). Activation of the supplementary motor areas enhances spinal reciprocal inhibition in healthy individuals. Brain Sci. 10, 587. doi: 10.3390/brainsci10090587. PMID: 32847117 PMC7565304

[B86] Hsu S. P. Lu C. F. Lin B. F. Tang C. W. Kuo I. J. Tsai Y. A. . (2023). Effects of bihemispheric transcranial direct current stimulation on motor recovery in subacute stroke patients: a double-blind, randomized sham-controlled trial. J. NeuroEng. Rehabil. 20, 27. doi: 10.1186/s12984-023-01153-4. PMID: 36849990 PMC9969953

[B87] Hsu T. Y. Tseng L. Y. Yu J. X. Kuo W. J. Hung D. L. Tzeng O. J. . (2011). Modulating inhibitory control with direct current stimulation of the superior medial frontal cortex. Neuroimage 56, 2249–2257. doi: 10.1016/j.neuroimage.2011.03.059. PMID: 21459149

[B88] Huang J. Bao C. Chen Y. Zhu W. Zhang K. Liu C. . (2025). Comparative efficacy and acceptability of non-invasive neuromodulation technologies and botulinum toxin injections for post-stroke spasticity and motor function: a network meta-analysis of randomised controlled trials. EClinicalMedicine 80, 103034. doi: 10.1016/j.eclinm.2024.103034. PMID: 39831129 PMC11741030

[B89] Huang L. Zhang J.-M. Bi Z.-T. Xiao J.-H. Wei J.-X. Huang J. . (2025). Effects of respiratory muscle training on respiratory function, exercise capacity, and quality of life in chronic stroke patients: a systematic review and meta-analysis. Front. Physiol. 16. doi: 10.3389/fphys.2025.1642262. PMID: 41049488 PMC12488600

[B90] Huimeng C. Xianglin W. Feng X. Hui L. Qiujie L. (2025). The effects of cognitive dual-task gait training and motor dual-task gait training on lower-limb coordination during walking in subacute stroke patients: A randomized controlled trial. Gait Posture 120, 17–24. doi: 10.1016/j.gaitpost.2025.03.021. PMID: 40179653

[B91] Huo C. Xu G. Sun A. Xie H. Hu X. Li W. . (2023). Cortical response induced by task-oriented training of the upper limb in subacute stroke patients as assessed by functional near-infrared spectroscopy. J. Biophotonics 16, e202200228. doi: 10.1002/jbio.202200228. PMID: 36222197

[B92] Huo C. Xu G. Xie H. Chen T. Shao G. Wang J. . (2024). Functional near-infrared spectroscopy in non-invasive neuromodulation. Neural Regener. Res. 19, 1517–1522. doi: 10.4103/1673-5374.387970. PMID: 38051894 PMC10883499

[B93] Hupfeld K. E. Ketcham C. J. Schneider H. D. (2017). Transcranial direct current stimulation (tDCS) to the supplementary motor area (SMA) influences performance on motor tasks. Exp. Brain Res. 235, 851–859. doi: 10.1007/s00221-016-4848-5. PMID: 27909747

[B94] Impey D. de la Salle S. Baddeley A. Knott V. (2017). Effects of an NMDA antagonist on the auditory mismatch negativity response to transcranial direct current stimulation. J. Psychopharmacol. 31, 614–624. doi: 10.1177/0269881116665336. PMID: 27624152

[B95] Ismail U. N. Yahya N. Manan H. A. (2024). Investigating functional connectivity related to stroke recovery: a systematic review. Brain Res. 1840, 149023. doi: 10.1016/j.brainres.2024.149023. PMID: 38815644

[B96] Jiang A. Wang Z. Song D. Zhang X. Guan M. Li X. . (2025). The application of ultrasound‐induced blood–brain barrier opening in neurology and immunology. Small 21, 2502699. doi: 10.1002/smll.202502699. PMID: 40519098

[B97] Jiménez de la Peña M. D. M. Gil-Robles S. Aracil C. Casado E. A. Rubio Alonso M. Martínez de Vega V. (2024). Postoperative reorganization of the supplementary motor area complex: a possible latent bihemispheric network. Clin. Neurol. Neurosurg. 246, 108586. doi: 10.1016/j.clineuro.2024.108586. PMID: 39378707

[B98] Jin Y. Bai X. Jiang B. Guo Z. Mu Q. (2022). Repetitive transcranial magnetic stimulation induces quantified functional and structural changes in subcortical stroke: a combined arterial spin labeling perfusion and diffusion tensor imaging study. Front. Hum. Neurosci. 16. doi: 10.3389/fnhum.2022.829688. PMID: 35463928 PMC9019060

[B99] Jung S. H. Park E. Kim J. H. Park B. A. Yu J. W. Kim A. R. . (2021). Effects of self rehabilitation video exercises (SAVE) on functional restorations in patients with subacute stroke. Healthc. (Basel) 9, 565. doi: 10.3390/healthcare9050565. PMID: 34064979 PMC8150768

[B100] Kahl C. K. Swansburg R. Kirton A. Pringsheim T. Wilcox G. Zewdie E. . (2021). Targeted interventions in Tourette's using advanced neuroimaging and stimulation (TITANS): study protocol for a double-blind, randomised controlled trial of transcranial magnetic stimulation (TMS) to the supplementary motor area in children with Tourette's syndrome. BMJ Open 11, e053156. doi: 10.1136/bmjopen-2021-053156. PMID: 34952879 PMC8712978

[B101] Karbe H. Thiel A. Weber-Luxenburger G. Herholz K. Kessler J. Heiss W. D. (1998). Brain plasticity in poststroke aphasia: what is the contribution of the right hemisphere? Brain Lang. 64, 215–230. doi: 10.1006/brln.1998.1961. PMID: 9710490

[B102] Kim S. Y. Hsu J. E. Husbands L. C. Kleim J. A. Jones T. A. (2018). Coordinated plasticity of synapses and astrocytes underlies practice-driven functional vicariation in peri-infarct motor cortex. J. Neurosci. 38, 93–107. doi: 10.1523/jneurosci.1295-17.2017. PMID: 29133435 PMC5761439

[B103] Kim Y. J. Ku J. Cho S. Kim H. J. Cho Y. K. Lim T. . (2014). Facilitation of corticospinal excitability by virtual reality exercise following anodal transcranial direct current stimulation in healthy volunteers and subacute stroke subjects. J. NeuroEng. Rehabil. 11, 124. doi: 10.1186/1743-0003-11-124. PMID: 25135003 PMC4148539

[B104] Kim W. S. Lee K. Kim S. Cho S. Paik N. J. (2019). Transcranial direct current stimulation for the treatment of motor impairment following traumatic brain injury. J. NeuroEng. Rehabil. 16, 14. doi: 10.1186/s12984-019-0489-9. PMID: 30683136 PMC6347832

[B105] Kim J. Lee S. H. Kim E. Kang S. R. Jo Y. J. Yun J. E. . (2026). Efficacy and safety of high-definition transcranial direct current stimulation combined with digital rehabilitation on upper limb function in stroke patients: study protocol for a randomized, double-blind, sham-controlled confirmatory trial. Trials 27, 131. doi: 10.1186/s13063-026-09461-5. PMID: 41668204 PMC12903231

[B106] Kim S. Y. Lee M. Y. Lee B. H. (2024). Effects of rehabilitation robot training on physical function, functional recovery, and daily living activities in patients with sub-acute stroke. Med. (Kaunas) 60, 811. doi: 10.3390/medicina60050811. PMID: 38792996 PMC11123305

[B107] Kim D. Y. Lim J. Y. Kang E. K. You D. S. Oh M. K. Oh B. M. . (2010). Effect of transcranial direct current stimulation on motor recovery in patients with subacute stroke. Am. J. Phys. Med. Rehabil. 89, 879–886. doi: 10.1097/PHM.0b013e3181f70aa7. PMID: 20962598

[B108] Kirimoto H. Ogata K. Onishi H. Oyama M. Goto Y. Tobimatsu S. (2011). Transcranial direct current stimulation over the motor association cortex induces plastic changes in ipsilateral primary motor and somatosensory cortices. Clin. Neurophysiol. 122, 777–783. doi: 10.1016/j.clinph.2010.09.025. PMID: 21074492

[B109] Klein F. Debener S. Witt K. Kranczioch C. (2022). fMRI-based validation of continuous-wave fNIRS of supplementary motor area activation during motor execution and motor imagery. Sci. Rep. 12, 3570. doi: 10.1038/s41598-022-06519-7. PMID: 35246563 PMC8897516

[B110] Klomjai W. Lackmy-Vallée A. Roche N. Pradat-Diehl P. Marchand-Pauvert V. Katz R. (2015). Repetitive transcranial magnetic stimulation and transcranial direct current stimulation in motor rehabilitation after stroke: an update. Ann. Phys. Rehabil. Med. 58, 220–224. doi: 10.1016/j.rehab.2015.05.006. PMID: 26272418

[B111] Komaitis S. Koutsarnakis C. Lani E. Kalamatianos T. Drosos E. Skandalakis G. P. . (2021). Deciphering the frontostriatal circuitry through the fiber dissection technique: direct structural evidence on the morphology and axonal connectivity of the fronto-caudate tract. J. Neurosurg. 135, 815–827. doi: 10.3171/2020.7.Jns201287. PMID: 33385993

[B112] Konrad C. Henningsen H. Bremer J. Mock B. Deppe M. Buchinger C. . (2002). Pattern of cortical reorganization in amyotrophic lateral sclerosis: a functional magnetic resonance imaging study. Exp. Brain Res. 143, 51–56. doi: 10.1007/s00221-001-0981-9. PMID: 11907690

[B113] Kulkarni R. Andraska E. McEnaney R. (2021). Structural remodeling of the extracellular matrix in arteriogenesis: a review. Front. Cardiovasc. Med. 8. doi: 10.3389/fcvm.2021.761007. PMID: 34805316 PMC8602576

[B114] Kwakkel G. Stinear C. Essers B. Munoz-Novoa M. Branscheidt M. Cabanas-Valdés R. . (2023). Motor rehabilitation after stroke: European Stroke Organisation (ESO) consensus-based definition and guiding framework. Eur. Stroke J. 8, 880–894. doi: 10.1177/23969873231191304. PMID: 37548025 PMC10683740

[B115] Lai M. H. Wang Y. F. Lu Y. Fu W. Zhang E. B. Ma H. L. . (2025). Case report: 40Hz multi-target transcranial alternating current stimulation combined with rehabilitation for post-stroke cognitive impairment. Front. Psychiatry 16. doi: 10.3389/fpsyt.2025.1682068. PMID: 41190302 PMC12580259

[B116] Larsen L. H. Zibrandtsen I. C. Wienecke T. Kjaer T. W. Langberg H. Nielsen J. B. . (2018). Modulation of task-related cortical connectivity in the acute and subacute phase after stroke. Eur. J. Neurosci. 47, 1024–1032. doi: 10.1111/ejn.13874. PMID: 29465793

[B117] Lazaridou A. Astrakas L. Mintzopoulos D. Khanchiceh A. Singhal A. Moskowitz M. . (2013). fMRI as a molecular imaging procedure for the functional reorganization of motor systems in chronic stroke. Mol. Med. Rep. 8, 775–779. doi: 10.3892/mmr.2013.1603. PMID: 23900349 PMC3782530

[B118] Lee H. J. Shin H. K. Shin Y. I. Kim J. H. Choi B. T. (2026). The fundamental mechanism of transcranial electrical stimulation in post-stroke rehabilitation. Front. Biosci. (Landmark Ed) 31, 46519. doi: 10.31083/fbl46519. PMID: 41609073

[B119] Lee S. H. Yoo Y. J. (2024). A literature review on optimal stimulation parameters of transcranial direct current stimulation for motor recovery after stroke. Brain Neurorehabil. 17, e24. doi: 10.12786/bn.2024.17.e24. PMID: 39649716 PMC11621672

[B120] Levin M. F. Berman S. Weiss N. Parmet Y. Baniña M. C. Frenkel-Toledo S. . (2023). ENHANCE proof-of-concept three-arm randomized trial: effects of reaching training of the hemiparetic upper limb restricted to the spasticity-free elbow range. Sci. Rep. 13, 22934. doi: 10.1038/s41598-023-49974-6. PMID: 38129527 PMC10739929

[B121] Li S. (2017). Spasticity, motor recovery, and neural plasticity after stroke. Front. Neurol. 8. doi: 10.3389/fneur.2017.00120. PMID: 28421032 PMC5377239

[B122] Li W. Cao P. Wei R. Wong D. W. (2025). Effect of sequential repetitive transcranial magnetic stimulation with bilateral arm training on the brain effective connectivity in chronic stroke. J. Biophotonics 18, e202400508. doi: 10.1002/jbio.202400508. PMID: 40035295

[B123] Li C. Chen Y. Tu S. Lin J. Lin Y. Xu S. . (2024). Dual-tDCS combined with sensorimotor training promotes upper limb function in subacute stroke patients: A randomized, double-blinded, sham-controlled study. CNS Neurosci. Ther. 30, e14530. doi: 10.1111/cns.14530. PMID: 37994674 PMC11017427

[B124] Li X. Ding X. He Y. Yi W. Zhu Y. Han W. . (2024). Ultrasound tissue engineering technology for regulating immune microenvironment. Adv. Funct. Mater. 34, 2400656. doi: 10.1002/adfm.202400656. PMID: 41859965

[B125] Li R. Li S. Roh J. Wang C. Zhang Y. (2020). Multimodal neuroimaging using concurrent EEG/fNIRS for poststroke recovery assessment: An exploratory study. Neurorehabil. Neural Repair 34, 1099–1110. doi: 10.1177/1545968320969937. PMID: 33190571

[B126] Li C. Wong Y. Langhammer B. Huang F. Du X. Wang Y. . (2022). A study of dynamic hand orthosis combined with unilateral task-oriented training in subacute stroke: A functional near-infrared spectroscopy case series. Front. Neurol. 13. doi: 10.3389/fneur.2022.907186. PMID: 36034313 PMC9410701

[B127] Li K. P. Wu J. J. Zhou Z. L. Xu D. S. Zheng M. X. Hua X. Y. . (2023). Noninvasive brain stimulation for neurorehabilitation in post-stroke patients. Brain Sci. 13, 451. doi: 10.3390/brainsci13030451. PMID: 36979261 PMC10046557

[B128] Li Y. Yu Z. Wu P. Chen J. (2021). The disrupted topological properties of structural networks showed recovery in ischemic stroke patients: a longitudinal design study. BMC Neurosci. 22, 47. doi: 10.1186/s12868-021-00652-1. PMID: 34340655 PMC8330082

[B129] Li X. Zhang H. Zhang W. Wu J. Dai L. Long N. . (2025). Neural mechanisms underlying the improvement of gait disturbances in stroke patients through robot-assisted gait training based on QEEG and fNIRS: a randomized controlled study. J. NeuroEng. Rehabil. 22, 136. doi: 10.1186/s12984-025-01656-2. PMID: 40533805 PMC12175367

[B130] Lin Y. Jiang Z. Zhan G. Su H. Kang X. Jia J. (2023). Brain network characteristics between subacute and chronic stroke survivors in active, imagery, passive movement task: A pilot study. Front. Neurol. 14. doi: 10.3389/fneur.2023.1143955. PMID: 37538258 PMC10395333

[B131] Lin J. Jin S. You Y. Liu J. Lu J. Shu Z. . (2025). EEG-fNIRS multilayer brain network analysis revealed functional neural reorganization of rTMS with motor training in stroke. IEEE Trans. Biomed. Eng. 73, 269–280. doi: 10.1109/tbme.2025.3580943. PMID: 40531644

[B132] Liu H. Cai W. Xu L. Li W. Qin W. (2019). Differential reorganization of SMA subregions after stroke: a subregional level resting-state functional connectivity study. Front. Hum. Neurosci. 13. doi: 10.3389/fnhum.2019.00468. PMID: 32184712 PMC7059000

[B133] Liu W. Cheng X. Rao J. Yu J. Lin Z. Wang Y. . (2024). Motor imagery therapy improved upper limb motor function in stroke patients with hemiplegia by increasing functional connectivity of sensorimotor and cognitive networks. Front. Hum. Neurosci. 18. doi: 10.3389/fnhum.2024.1295859. PMID: 38439937 PMC10910033

[B134] Liu F. T. Lu J. Y. Sun Y. M. Li L. Yang Y. J. Zhao J. . (2023). Dopaminergic dysfunction and glucose metabolism characteristics in Parkin-induced early-onset Parkinson's disease compared to genetically undetermined early-onset Parkinson's disease. Phenomics 3, 22–33. doi: 10.1007/s43657-022-00077-8. PMID: 36939793 PMC9883374

[B135] Liu J. Wang C. (2022). Microstructure and genetic polymorphisms: Role in motor rehabilitation after subcortical stroke. Front. Aging Neurosci. 14. doi: 10.3389/fnagi.2022.813756. PMID: 35177977 PMC8843845

[B136] Liu J. Wang C. Qin W. Ding H. Guo J. Han T. . (2020). Corticospinal fibers with different origins impact motor outcome and brain after subcortical stroke. Stroke 51, 2170–2178. doi: 10.1161/strokeaha.120.029508. PMID: 32568657

[B137] Liu Z. Yu Q. Zhou F. Yu M. Shu H. Zhu M. . (2025). Repetitive transcranial magnetic stimulation and constraint-induced movement therapy combined in the treatment of post-stroke movement disorders: a narrative review. Front. Hum. Neurosci. 19. doi: 10.3389/fnhum.2025.1578258. PMID: 40260173 PMC12009840

[B138] Longo V. Barbati S. A. Re A. Paciello F. Bolla M. Rinaudo M. . (2022). Transcranial direct current stimulation enhances neuroplasticity and accelerates motor recovery in a stroke mouse model. Stroke 53, 1746–1758. doi: 10.1161/strokeaha.121.034200. PMID: 35291824

[B139] Lv L. Cheng X. Yang J. Chen X. Ni J. (2023). Novel role for non-invasive neuromodulation techniques in central respiratory dysfunction. Front. Neurosci. 17. doi: 10.3389/fnins.2023.1226660. PMID: 37680969 PMC10480838

[B140] Ma Y. Xie D. Yu Y. Yao K. Zhang S. Li Q. . (2025). Differences in brain activation and connectivity during unaffected hand exercise in subacute and convalescent stroke patients. Neuroscience 565, 10–18. doi: 10.1016/j.neuroscience.2024.11.038. PMID: 39561956

[B141] Mahjoub Y. Szejko N. Gan L. S. Adeoti J. A. Nitsche M. A. Vicario C. M. . (2025). Randomized controlled trial of transcranial direct current stimulation over the supplementary motor area in Tourette syndrome. Mov. Disord. Clin. Pract. 12, 313–324. doi: 10.1002/mdc3.14285. PMID: 39614604 PMC11952956

[B142] Malerba P. Straudi S. Fregni F. Bazhenov M. Basaglia N. (2017). Using biophysical models to understand the effect of tDCS on neurorehabilitation: Searching for optimal covariates to enhance poststroke recovery. Front. Neurol. 8. doi: 10.3389/fneur.2017.00058. PMID: 28280482 PMC5322214

[B143] Manganotti P. Acler M. Formaggio E. Avesani M. Milanese F. Baraldo A. . (2010). Changes in cerebral activity after decreased upper-limb hypertonus: an EMG-fMRI study. Magn. Reson. Imaging 28, 646–652. doi: 10.1016/j.mri.2009.12.023. PMID: 20117894

[B144] Manji A. Amimoto K. Matsuda T. Wada Y. Inaba A. Ko S. (2018). Effects of transcranial direct current stimulation over the supplementary motor area body weight-supported treadmill gait training in hemiparetic patients after stroke. Neurosci. Lett. 662, 302–305. doi: 10.1016/j.neulet.2017.10.049. PMID: 29107706

[B145] Matsunaga K. Maruyama A. Fujiwara T. Nakanishi R. Tsuji S. Rothwell J. C. (2005). Increased corticospinal excitability after 5 Hz rTMS over the human supplementary motor area. J. Physiol. 562, 295–306. doi: 10.1113/jphysiol.2004.070755. PMID: 15513947 PMC1665472

[B146] Mazzoleni S. Duret C. Grosmaire A. G. Battini E. (2017). Combining upper limb robotic rehabilitation with other therapeutic approaches after stroke: Current status, rationale, and challenges. BioMed. Res. Int. 2017, 8905637. doi: 10.1155/2017/8905637. PMID: 29057269 PMC5615953

[B147] McClure L. A. Szychowski J. M. Benavente O. Hart R. G. Coffey C. S. (2016). A post hoc evaluation of a sample size re-estimation in the Secondary Prevention of Small Subcortical Strokes study. Clin. Trials 13, 537–544. doi: 10.1177/1740774516643689. PMID: 27094488 PMC5025324

[B148] Mehrabi S. Flores-Sandoval C. Fleet J. L. Dukelow S. P. Bateman E. A. Teasell R. (2025). Time post-stroke and upper extremity stroke motor recovery rehabilitation: A meta-analysis. Neurorehabil. Neural Repair 39, 945–953. doi: 10.1177/15459683251356975. PMID: 40698597 PMC12531395

[B149] Meijer R. Ihnenfeldt D. S. de Groot I. J. Van Limbeek J. Vermeulen M. De Haan R. J. (2003). Prognostic factors for ambulation and activities of daily living in the subacute phase after stroke. A systematic review of the literature. Clin. Rehabil. 17, 119–129. doi: 10.1191/0269215503cr585oa. PMID: 12625651

[B150] Mekbib D. B. Zhao Z. Wang J. Xu B. Zhang L. Cheng R. . (2020). Proactive motor functional recovery following immersive virtual reality-based limb mirroring therapy in patients with subacute stroke. Neurotherapeutics 17, 1919–1930. doi: 10.1007/s13311-020-00882-x. PMID: 32671578 PMC7851292

[B151] Meng H. Houston M. Zhang Y. Li S. (2024). Exploring the prospects of transcranial electrical stimulation (tES) as a therapeutic intervention for post-stroke motor recovery: A narrative review. Brain Sci. 14, 322. doi: 10.3390/brainsci14040322. PMID: 38671974 PMC11047964

[B152] Miranda de Aquino Miranda J. Sousa de Andrade P. H. Henrique M. Henrique de Souza Fonseca B. Bazan R. Sande de Souza L. A. P. . (2025). The effect of transcranial direct current stimulation combined with task-specific training on spatio-temporal gait parameters and functional mobility in individuals with stroke: a systematic review and meta-analysis. Top. Stroke Rehabil. 32, 438–457. doi: 10.1080/10749357.2024.2411878. PMID: 39470996

[B153] Mitsutake T. Imura T. Hori T. Sakamoto M. Tanaka R. (2021). Effects of combining online anodal transcranial direct current stimulation and gait training in stroke patients: A systematic review and meta-analysis. Front. Hum. Neurosci. 15. doi: 10.3389/fnhum.2021.782305. PMID: 34955795 PMC8708562

[B154] Miyaguchi S. Inukai Y. Takahashi R. Miyashita M. Matsumoto Y. Otsuru N. . (2020). Effects of stimulating the supplementary motor area with a transcranial alternating current for bimanual movement performance. Behav. Brain Res. 393, 112801. doi: 10.1016/j.bbr.2020.112801. PMID: 32652107

[B155] Nakahara H. Doya K. Hikosaka O. (2001). Parallel cortico-basal ganglia mechanisms for acquisition and execution of visuomotor sequences - a computational approach. J. Cognit. Neurosci. 13, 626–647. doi: 10.1162/089892901750363208. PMID: 11506661

[B156] Narayana S. Zhang W. Rogers W. Strickland C. Franklin C. Lancaster J. L. . (2014). Concurrent TMS to the primary motor cortex augments slow motor learning. Neuroimage 85 Pt 3, 971–984. doi: 10.1016/j.neuroimage.2013.07.024. PMID: 23867557 PMC4331120

[B157] Nasrallah F. A. Mohamed A. Z. Yap H. K. Lai H. S. Yeow C. H. Lim J. H. (2021). Effect of proprioceptive stimulation using a soft robotic glove on motor activation and brain connectivity in stroke survivors. J. Neural Eng. 18. doi: 10.1088/1741-2552/ac456c. PMID: 34933283

[B158] Neige C. Vassiliadis P. Ali Zazou A. Dricot L. Lebon F. Brees T. . (2023). Connecting the dots: harnessing dual-site transcranial magnetic stimulation to quantify the causal influence of medial frontal areas on the motor cortex. Cereb. Cortex 33, 11339–11353. doi: 10.1093/cercor/bhad370. PMID: 37804253

[B159] Nie J. Yang X. Tang Q. Shen Q. Li S. (2016). Willed-movement training reduces brain damage and enhances synaptic plasticity related proteins synthesis after focal ischemia. Brain Res. Bull. 120, 90–96. doi: 10.1016/j.brainresbull.2015.11.004. PMID: 26556240

[B160] Nomura T. Kirimoto H. (2018). Anodal transcranial direct current stimulation over the supplementary motor area improves anticipatory postural adjustments in older adults. Front. Hum. Neurosci. 12. doi: 10.3389/fnhum.2018.00317. PMID: 30123118 PMC6086140

[B161] Okabe N. Shiromoto T. Himi N. Lu F. Maruyama-Nakamura E. Narita K. . (2016). Neural network remodeling underlying motor map reorganization induced by rehabilitative training after ischemic stroke. Neuroscience 339, 338–362. doi: 10.1016/j.neuroscience.2016.10.008. PMID: 27725217

[B162] Oostra K. M. Oomen A. Vanderstraeten G. Vingerhoets G. (2015). Influence of motor imagery training on gait rehabilitation in sub-acute stroke: A randomized controlled trial. J. Rehabil. Med. 47, 204–209. doi: 10.2340/16501977-1908. PMID: 25403275

[B163] Padoa-Schioppa C. Li C. S. Bizzi E. (2002). Neuronal correlates of kinematics-to-dynamics transformation in the supplementary motor area. Neuron 36, 751–765. doi: 10.1016/s0896-6273(02)01028-0. PMID: 12441062

[B164] Pantano P. Formisano R. Ricci M. Di Piero V. Sabatini U. Di Pofi B. . (1996). Motor recovery after stroke. Morphological and functional brain alterations. Brain 119, 1849–1857. doi: 10.1093/brain/119.6.1849. PMID: 9009992

[B165] Papoutsi M. Weiskopf N. Langbehn D. Reilmann R. Rees G. Tabrizi S. J. (2018). Stimulating neural plasticity with real-time fMRI neurofeedback in Huntington's disease: a proof of concept study. Hum. Brain Mapp. 39, 1339–1353. doi: 10.1002/hbm.23921. PMID: 29239063 PMC5838530

[B166] Parsons N. Irimia A. Amgalan A. Ugon J. Morgan K. Shelyag S. . (2023). Structural-functional connectivity bandwidth predicts processing speed in mild traumatic brain injury: a multiplex network analysis. NeuroImage Clin. 38, 103428. doi: 10.1016/j.nicl.2023.103428. PMID: 37167841 PMC10196722

[B167] Páscoa Dos Santos F. de la Torre Costa J. Maier M. Rubio Ballester B. Engracia Perez M. Buxó X. . (2026). Preventing slowing down of alpha rhythms in stroke patients through modulation of cortical excitatory-inhibitory balance: a randomized controlled trial. J. NeuroEng. Rehabil. 23, 102. doi: 10.1186/s12984-026-01906-x. PMID: 41703634 PMC13014810

[B168] Pekala K. Michalak A. Kruk-Slomka M. Budzynska B. Biala G. (2018). Impacts of cannabinoid receptor ligands on nicotine- and chronic mild stress-induced cognitive and depression-like effects in mice. Behav. Brain Res. 347, 167–174. doi: 10.1016/j.bbr.2018.03.019. PMID: 29551733

[B169] Peng Q. C. Yin L. Cao Y. (2021). Effectiveness of virtual reality in the rehabilitation of motor function of patients with subacute stroke: A meta-analysis. Front. Neurol. 12. doi: 10.3389/fneur.2021.639535. PMID: 34025553 PMC8131676

[B170] Perez M. A. Tanaka S. Wise S. P. Sadato N. Tanabe H. C. Willingham D. T. . (2007). Neural substrates of intermanual transfer of a newly acquired motor skill. Curr. Biol. 17, 1896–1902. doi: 10.1016/j.cub.2007.09.058. PMID: 17964167

[B171] Phipps M. S. Cronin C. A. (2020). Management of acute ischemic stroke. BMJ 368, l6983. doi: 10.1136/bmj.l6983. PMID: 32054610

[B172] Pinson H. Van Lerbeirghe J. Vanhauwaert D. Van Damme O. Hallaert G. Kalala J. P. (2022). The supplementary motor area syndrome: a neurosurgical review. Neurosurg. Rev. 45, 81–90. doi: 10.1007/s10143-021-01566-6. PMID: 33993354

[B173] Pirovano I. Antonacci Y. Mastropietro A. Bara C. Sparacino L. Guanziroli E. . (2023). Rehabilitation modulates high-order interactions among large-scale brain networks in subacute stroke. IEEE Trans. Neural Syst. Rehabil. Eng. 31, 4549–4560. doi: 10.1109/tnsre.2023.3332114. PMID: 37955999

[B174] Pollok B. Makhloufi H. Butz M. Gross J. Timmermann L. Wojtecki L. . (2009). Levodopa affects functional brain networks in Parkinsonian resting tremor. Mov. Disord. 24, 91–98. doi: 10.1002/mds.22318. PMID: 18823037

[B175] Qi S. Cao L. Wang Q. Sheng Y. Yu J. Liang Z. (2024). The physiological mechanisms of transcranial direct current stimulation to enhance motor performance: A narrative review. Biol. (Basel) 13, 790. doi: 10.3390/biology13100790. PMID: 39452099 PMC11504865

[B176] Qi L. Wang C. Deng L. Pan J. J. Suo Q. Wu S. . (2024). Low-intensity focused ultrasound stimulation promotes stroke recovery via astrocytic HMGB1 and CAMK2N1 in mice. Stroke Vasc. Neurol. 9, 505–518. doi: 10.1136/svn-2023-002614. PMID: 38191183 PMC11732924

[B177] Quandt F. Bönstrup M. Schulz R. Timmermann J. E. Mund M. Wessel M. J. . (2019). The functional role of beta-oscillations in the supplementary motor area during reaching and grasping after stroke: a question of structural damage to the corticospinal tract. Hum. Brain Mapp. 40, 3091–3101. doi: 10.1002/hbm.24582. PMID: 30927325 PMC6865486

[B178] Rehme A. K. Eickhoff S. B. Wang L. E. Fink G. R. Grefkes C. (2011). Dynamic causal modeling of cortical activity from the acute to the chronic stage after stroke. Neuroimage 55, 1147–1158. doi: 10.1016/j.neuroimage.2011.01.014. PMID: 21238594 PMC8053821

[B179] Richards L. Hanson C. Wellborn M. Sethi A. (2008). Driving motor recovery after stroke. Top. Stroke Rehabil. 15, 397–411. doi: 10.1310/tsr1505-397. PMID: 19008201

[B180] Rogasch N. C. Fitzgerald P. B. (2013). Assessing cortical network properties using TMS-EEG. Hum. Brain Mapp. 34, 1652–1669. doi: 10.1002/hbm.22016. PMID: 22378543 PMC6870446

[B181] Romero J. P. Martínez-Benito A. de Noreña D. Hurtado-Martínez A. Sánchez-Cuesta F. J. González-Zamorano Y. . (2024). Combined non-invasive neuromodulation using transcranial direct current stimulation, motor imagery and action observation for motor, cognitive and functional recovery in cortico-basal degeneration: a single case study. Excli J. 23, 714–726. doi: 10.17179/excli2024-7027. PMID: 38887394 PMC11180953

[B182] Ruas C. V. Carlos B. M. Feitosa S. Silva M. V. Vazquez P. Pontes L. L. . (2025). The effects of transcranial direct current stimulation during extended reality exercises for cortical, neuromuscular, and clinical recovery of stroke survivors. Neural Plast. 2025, 5688648. doi: 10.1155/np/5688648. PMID: 40697356 PMC12283194

[B183] Ruddy K. L. Leemans A. Woolley D. G. Wenderoth N. Carson R. G. (2017). Structural and functional cortical connectivity mediating cross education of motor function. J. Neurosci. 37, 2555–2564. doi: 10.1523/jneurosci.2536-16.2017. PMID: 28154150 PMC5354316

[B184] Sadler C. M. Kami A. T. Nantel J. Carlsen A. N. (2021). Transcranial direct current stimulation of supplementary motor area improves upper limb kinematics in Parkinson's disease. Clin. Neurophysiol. 132, 2907–2915. doi: 10.1016/j.clinph.2021.06.031. PMID: 34412968

[B185] Sale P. Franceschini M. Mazzoleni S. Palma E. Agosti M. Posteraro F. (2014). Effects of upper limb robot-assisted therapy on motor recovery in subacute stroke patients. J. NeuroEng. Rehabil. 11, 104. doi: 10.1186/1743-0003-11-104. PMID: 24946799 PMC4074149

[B186] Sánchez-Cuesta F. J. Arroyo-Ferrer A. González-Zamorano Y. Vourvopoulos A. Badia S. B. I. Figuereido P. . (2021). Clinical effects of immersive multimodal BCI-VR training after bilateral neuromodulation with rTMS on upper limb motor recovery after stroke. A study protocol for a randomized controlled trial. Med. (Kaunas) 57, 736. doi: 10.3390/medicina57080736. PMID: 34440942 PMC8401798

[B187] Saur D. Lange R. Baumgaertner A. Schraknepper V. Willmes K. Rijntjes M. . (2006). Dynamics of language reorganization after stroke. Brain 129, 1371–1384. doi: 10.1093/brain/awl090. PMID: 16638796

[B188] Scho S. Brüchle W. Schneefeld J. Rosenkranz K. (2024). Enhancing neuroplasticity in major depression: a novel 10 Hz-rTMS protocol is more effective than iTBS. J. Affect. Disord. 367, 109–117. doi: 10.1016/j.jad.2024.08.166. PMID: 39187195

[B189] Sebastianelli L. Versace V. Martignago S. Brigo F. Trinka E. Saltuari L. . (2017). Low-frequency rTMS of the unaffected hemisphere in stroke patients: A systematic review. Acta Neurol. Scand. 136, 585–605. doi: 10.1111/ane.12773. PMID: 28464421

[B190] Sharp D. J. Beckmann C. F. Greenwood R. Kinnunen K. M. Bonnelle V. De Boissezon X. . (2011). Default mode network functional and structural connectivity after traumatic brain injury. Brain 134, 2233–2247. doi: 10.1093/brain/awr175. PMID: 21841202

[B191] Shen Q. R. Hu M. T. Feng W. Li K. P. Wang W. (2022). Narrative review of noninvasive brain stimulation in stroke rehabilitation. Med. Sci. Monit. 28, e938298. doi: 10.12659/msm.938298. PMID: 36457205 PMC9724451

[B192] Shibasaki H. (2012). Cortical activities associated with voluntary movements and involuntary movements. Clin. Neurophysiol. 123, 229–243. doi: 10.1016/j.clinph.2011.07.042. PMID: 21906995

[B193] Shin J. An H. Yang S. Park C. Lee Y. You S. J. H. (2022). Comparative effects of passive and active mode robot-assisted gait training on brain and muscular activities in sub-acute and chronic stroke. NeuroRehabilitation 51, 51–63. doi: 10.3233/nre-210304. PMID: 35311717

[B194] Shin J. An H. Yang S. Park C. Lee Y. You S. J. H. (2023). Erratum to: Comparative effects of passive and active mode robot-assisted gait training on brain and muscular activities in sub-acute and chronic stroke. NeuroRehabilitation 53, 417. doi: 10.3233/nre-230004. PMID: 37270829

[B195] Shirota Y. Ohtsu H. Hamada M. Enomoto H. Ugawa Y. (2013). Supplementary motor area stimulation for Parkinson disease: a randomized controlled study. Neurology 80, 1400–1405. doi: 10.1212/WNL.0b013e31828c2f66. PMID: 23516319

[B196] Singh N. Saini M. Kumar N. Srivastava M. V. P. Mehndiratta A. (2021). Evidence of neuroplasticity with robotic hand exoskeleton for post-stroke rehabilitation: a randomized controlled trial. J. NeuroEng. Rehabil. 18, 76. doi: 10.1186/s12984-021-00867-7. PMID: 33957937 PMC8101163

[B197] Soltani P. Morovati Z. Yousefi Afrashteh M. Fink-Lamotte J. Alizadehgoradel J. (2026). Targeting the prefrontal-supplementary motor network with online and offline tDCS to modulate disgust: A single-blind and sham-controlled study. Behav. Brain Res. 504, 116077. doi: 10.1016/j.bbr.2026.116077. PMID: 41638260

[B198] Stinear C. M. Byblow W. D. (2014). Predicting and accelerating motor recovery after stroke. Curr. Opin. Neurol. 27, 624–630. doi: 10.1097/wco.0000000000000153. PMID: 25364953

[B199] Swarnakar R. (2025). Precision rehabilitation through mathematical modeling: A new frontier in medicine. Cureus 17, e86965. doi: 10.7759/cureus.86965. PMID: 40734876 PMC12306515

[B200] Tabari F. Patron C. Cryer H. Johari K. (2024). HD-tDCS over left supplementary motor area differentially modulated neural correlates of motor planning for speech vs. limb movement. Int. J. Psychophysiol. 201, 112357. doi: 10.1016/j.ijpsycho.2024.112357. PMID: 38701898

[B201] Takeuchi N. Izumi S. (2012). Noninvasive brain stimulation for motor recovery after stroke: mechanisms and future views. Stroke Res. Treat. 2012, 584727. doi: 10.1155/2012/584727. PMID: 23050198 PMC3463193

[B202] Takeuchi N. Izumi S. (2013). Rehabilitation with poststroke motor recovery: a review with a focus on neural plasticity. Stroke Res. Treat. 2013, 128641. doi: 10.1155/2013/128641. PMID: 23738231 PMC3659508

[B203] Tanaka N. Matsushita S. Sonoda Y. Maruta Y. Fujitaka Y. Sato M. . (2019). Effect of stride management assist gait training for poststroke hemiplegia: A single center, open-label, randomized controlled trial. J. Stroke Cerebrovasc. Dis. 28, 477–486. doi: 10.1016/j.jstrokecerebrovasdis.2018.10.025. PMID: 30420315

[B204] Tanji J. (2001). Sequential organization of multiple movements: involvement of cortical motor areas. Annu. Rev. Neurosci. 24, 631–651. doi: 10.1146/annurev.neuro.24.1.631. PMID: 11520914

[B205] Torrisi M. Maggio M. G. De Cola M. C. Zichittella C. Carmela C. Porcari B. . (2021). Beyond motor recovery after stroke: The role of hand robotic rehabilitation plus virtual reality in improving cognitive function. J. Clin. Neurosci. 92, 11–16. doi: 10.1016/j.jocn.2021.07.053. PMID: 34509235

[B206] Umesawa Y. Atsumi T. Chakrabarty M. Fukatsu R. Ide M. (2020). GABA concentration in the left ventral premotor cortex associates with sensory hyper-responsiveness in autism spectrum disorders without intellectual disability. Front. Neurosci. 14. doi: 10.3389/fnins.2020.00482. PMID: 32508576 PMC7248307

[B207] Vajda F. J. (2002). Neuroprotection and neurodegenerative disease. J. Clin. Neurosci. 9, 4–8. doi: 10.1054/jocn.2001.1027. PMID: 11749009

[B208] van Assche M. Dirren E. Bourgeois A. Kleinschmidt A. Richiardi J. Carrera E. (2021). Periinfarct rewiring supports recovery after primary motor cortex stroke. J. Cereb. Blood Flow Metab. 41, 2174–2184. doi: 10.1177/0271678x211002968. PMID: 33757315 PMC8392854

[B209] Veldema J. Gharabaghi A. (2022). Non-invasive brain stimulation for improving gait, balance, and lower limbs motor function in stroke. J. NeuroEng. Rehabil. 19, 84. doi: 10.1186/s12984-022-01062-y. PMID: 35922846 PMC9351139

[B210] Vergani F. Ghimire P. Rajashekhar D. Dell'acqua F. Lavrador J. P. (2021). Superior longitudinal fasciculus (SLF) I and II: an anatomical and functional review. J. Neurosurg. Sci. 65, 560–565. doi: 10.23736/s0390-5616.21.05327-3. PMID: 33940781

[B211] Vergani F. Lacerda L. Martino J. Attems J. Morris C. Mitchell P. . (2014). White matter connections of the supplementary motor area in humans. J. Neurol. Neurosurg. Psychiatry 85, 1377–1385. doi: 10.1136/jnnp-2013-307492. PMID: 24741063

[B212] Vignozzi L. Macchi F. Montagni E. Pasquini M. Martello A. Minetti A. . (2025). Combining gamma neuromodulation and robotic rehabilitation after a stroke restores parvalbumin interneuron dynamics and improves motor recovery in mice. PloS Biol. 23, e3002806. doi: 10.1371/journal.pbio.3002806. PMID: 41086129 PMC12520394

[B213] Wang J. Gao Y. Jia X. Qin Z. Song J. Ke X. . (2026). Effects of motor imagery training based on near-infrared functional brain imaging on upper limb function in stroke patients: A randomized controlled trial. Brain Topogr. 39, 16. doi: 10.1007/s10548-025-01165-4. PMID: 41484476

[B214] Wang X. Liang X. Ku Y. Zhan Y. Song R. (2025). Effective motor skill learning induces inverted-U load-dependent activation in contralateral pre-motor and supplementary motor area. Hum. Brain Mapp. 46, e70208. doi: 10.1002/hbm.70208. PMID: 40186523 PMC11971689

[B215] Wang Q. L. Sun Y. X. Zhu L. Zhu S. Z. Geng A. Y. Xu S. . (2025). Effects of virtual reality upper limb rehabilitation robot on brain network in stroke patients with hemiplegia. Chin. J. Rehabil. Med. 40, 995–1001. doi: 10.3969/j.issn.1001-1242.2025.07.004. PMID: 35900448

[B216] Wang L. Zhang Y. Zhang J. Sang L. Li P. Yan R. . (2019). Aging changes effective connectivity of motor networks during motor execution and motor imagery. Front. Aging Neurosci. 11. doi: 10.3389/fnagi.2019.00312. PMID: 31824297 PMC6881270

[B217] Wang D. Zhou J. Huang Y. Meng Q. (2025). Effect of parallel cognitive-motor training tasks on hemodynamic responses in robot-assisted rehabilitation. Brain Connect. 15, 98–111. doi: 10.1089/brain.2024.0043. PMID: 39973310

[B218] Westlake K. P. Hinkley L. B. Bucci M. Guggisberg A. G. Byl N. Findlay A. M. . (2012). Resting state α-band functional connectivity and recovery after stroke. Exp. Neurol. 237, 160–169. doi: 10.1016/j.expneurol.2012.06.020. PMID: 22750324 PMC3646713

[B219] Wong W. W. Chan S. T. Tang K. W. Meng F. Tong K. Y. (2013). Neural correlates of motor impairment during motor imagery and motor execution in sub-cortical stroke. Brain Inj. 27, 651–663. doi: 10.3109/02699052.2013.771796. PMID: 23514275

[B220] Wu J. Cheng H. Zhang J. Bai Z. Cai S. (2021). The modulatory effects of bilateral arm training (BAT) on the brain in stroke patients: a systematic review. Neurol. Sci. 42, 501–511. doi: 10.1007/s10072-020-04854-z. PMID: 33180209

[B221] Wu Y. Tian C. Yu Z. Liu Z. Wu H. Ming J. . (2025). Structural and functional disconnections in non-acute post-stroke patients. Front. Neurol. 16. doi: 10.3389/fneur.2025.1542292. PMID: 40635710 PMC12237644

[B222] Xia W. Dai R. Xu X. Huai B. Bai Z. Zhang J. . (2022). Cortical mapping of active and passive upper limb training in stroke patients and healthy people: a functional near-infrared spectroscopy study. Brain Res. 1788, 147935. doi: 10.1016/j.brainres.2022.147935. PMID: 35500604

[B223] Xing Y. Bai Y. (2020). A review of exercise-induced neuroplasticity in ischemic stroke: Pathology and mechanisms. Mol. Neurobiol. 57, 4218–4231. doi: 10.1007/s12035-020-02021-1. PMID: 32691303

[B224] Xiong F. Liao X. Xiao J. Bai X. Huang J. Zhang B. . (2022). Emerging limb rehabilitation therapy after post-stroke motor recovery. Front. Aging Neurosci. 14. doi: 10.3389/fnagi.2022.863379. PMID: 35401147 PMC8984121

[B225] Xu Y. Zhang S. Mi J. Lu X. Wang Q. Fan R. . (2025). Modulatory effects of transcranial magneto-acoustic stimulation on behavior and corticostriatal transmission of oscillatory activity in a mouse model of Parkinson's disease induced by MPTP. Exp. Neurol. 392, 115352. doi: 10.1016/j.expneurol.2025.115352. PMID: 40543744

[B226] Yamashita K. Ida R. Koganemaru S. Horiba M. Nojima I. Mima T. . (2025). A pilot study on simultaneous stimulation of the primary motor cortex and supplementary motor area using gait-synchronized rhythmic brain stimulation to improve gait variability in post-stroke hemiparetic patients. Front. Hum. Neurosci. 19. doi: 10.3389/fnhum.2025.1618758. PMID: 41041019 PMC12484017

[B227] Yang H. E. Kyeong S. Lee S. H. Lee W. J. Ha S. W. Kim S. M. . (2017). Structural and functional improvements due to robot-assisted gait training in the stroke-injured brain. Neurosci. Lett. 637, 114–119. doi: 10.1016/j.neulet.2016.11.039. PMID: 27884739

[B228] Yang S. Li Y. Zhang F. Verheyden G. Chen Z. Li Y. . (2026). Investigating repetitive transcranial magnetic stimulation-induced interhemispheric changes in stroke: A transcranial magnetic stimulation and fNIRS study. Neurophotonics 13, S13010. doi: 10.1117/1.NPh.13.S1.S13010. PMID: 41674534 PMC12890182

[B229] Yoo Y. J. Park H. J. Kim T. Y. Yoon M. J. Oh H. M. Lee Y. J. . (2022). MRI-based personalized transcranial direct current stimulation to enhance the upper limb function in patients with stroke: Study protocol for a double-blind randomized controlled trial. Brain Sci. 12, 1673. doi: 10.3390/brainsci12121673. PMID: 36552133 PMC9775341

[B230] Yoo W. K. You S. H. Ko M. H. Tae Kim S. Park C. H. Park J. W. . (2008). High frequency rTMS modulation of the sensorimotor networks: Behavioral changes and fMRI correlates. Neuroimage 39, 1886–1895. doi: 10.1016/j.neuroimage.2007.10.035. PMID: 18086536

[B231] Yuan K. Wang X. Chen C. Lau C. C. Chu W. C. Tong R. K. (2020). Interhemispheric functional reorganization and its structural base after BCI-guided upper-limb training in chronic stroke. IEEE Trans. Neural Syst. Rehabil. Eng. 28, 2525–2536. doi: 10.1109/tnsre.2020.3027955. PMID: 32997632

[B232] Zaehle T. Rach S. Herrmann C. S. (2010). Transcranial alternating current stimulation enhances individual alpha activity in human EEG. PloS One 5, e13766. doi: 10.1371/journal.pone.0013766. PMID: 21072168 PMC2967471

[B233] Zhang J. Lu C. Wu X. Nie D. Yu H. (2021). Neuroplasticity of acupuncture for stroke: An evidence-based review of MRI. Neural Plast. 2021, 2662585. doi: 10.1155/2021/2662585. PMID: 34456996 PMC8397547

[B234] Zhang T. Pan N. Wang Y. Liu C. Hu S. (2021). Transcranial focused ultrasound neuromodulation: A review of the excitatory and inhibitory effects on brain activity in human and animals. Front. Hum. Neurosci. 15. doi: 10.3389/fnhum.2021.749162. PMID: 34650419 PMC8507972

[B235] Zhang J. Wang M. Chen H. Fan S. Sun R. Wang L. . (2025). Modulation of cerebral cortex activity by acupuncture combined with continuous theta-burst stimulation in post-stroke upper limb spasticity: an fNIRS study. Front. Neurol. 16. doi: 10.3389/fneur.2025.1542489. PMID: 40529436 PMC12171123

[B236] Zhang Y. Zhang Y. Zheng B. Chen S. Yu H. Dai L. . (2025). The effects of combining anodal transcranial direct current stimulation with robot-assisted gait training on lower limb motor function and the motor cortex regulation of stroke patients. J. NeuroEng. Rehabil. 22, 230. doi: 10.1186/s12984-025-01731-8. PMID: 41163056 PMC12574300

[B237] Zhao L. Chen L. Wang Q. Li X. Li S. Wan C. (2025). The effect of rTMS intervention with different targets on neural remodeling in stroke patients: A randomized controlled trial. Front. Neurol. 16. doi: 10.3389/fneur.2025.1539393. PMID: 40746643 PMC12310651

[B238] Zhao L. Chen L. Wang C. Li S. Wan C. (2024). Effects of repetitive transcranial magnetic stimulation at different targets on brain function in stroke patients: a randomized controlled trial. Front. Neurol. 15. doi: 10.3389/fneur.2024.1454220. PMID: 39403265 PMC11471684

[B239] Zheng J. Shi P. Fan M. Liang S. Li S. Yu H. (2021). Effects of passive and active training modes of upper-limb rehabilitation robot on cortical activation: A functional near-infrared spectroscopy study. Neuroreport 32, 479–488. doi: 10.1097/wnr.0000000000001615. PMID: 33788815

[B240] Zheng Y. Zhang J. Yang Y. Xu M. (2025). Neural representation of sensorimotor features in language-motor areas during auditory and visual perception. Commun. Biol. 8, 41. doi: 10.1038/s42003-025-07466-5. PMID: 39799186 PMC11724955

